# When the desert dries: rainfall drives conflicts and conservation challenges for onager (*Equus hemionus onager*)

**DOI:** 10.1093/jmammal/gyag017

**Published:** 2026-05-01

**Authors:** Saeideh Esmaeili, Kathryn A Schoenecker, Mahmoud-Reza Hemami, Petra Kaczensky, Chris Walzer, Jacob R Goheen

**Affiliations:** Natural Resource Ecology Laboratory, Warner College of Natural Resources, Colorado State University, 1231 Libbie Coy Way, Fort Collins, CO 80523-1499, United States; U.S. Geological Survey, Fort Collins Science Center, 2150 Centre Ave # C, Fort Collins, CO 80526, United States; Ecosystem Science and Sustainability, Colorado State University, 1231 Libbie Coy Wy, Fort Collins, CO 80521, United States; Department of Natural Resources, Isfahan University of Technology, Isfahan Province, Khomeyni Shahr, Daneshgah e Sanati Hwy, PG9G+39R, Isfahan, Iran; Department of Forestry and Wildlife Management, Inland Norway University of Applied Sciences, Anne Evenstads veg 80, 2480, Koppang, Norway; Research Institute of Wildlife Ecology, University of Veterinary Medicine Vienna, Savoyenstraße 1, 1160 Vienna, Austria; Department of Terrestrial Biodiversity, Norwegian Institute for Nature Research, Høgskoleringen 9, 7034 Trondheim, Norway; Research Institute of Wildlife Ecology, University of Veterinary Medicine Vienna, Savoyenstraße 1, 1160 Vienna, Austria; Wildlife Conservation Society, 2300 Southern Boulevard. Bronx, New York, 10460, United States; Department of Natural Resource Ecology & Management, Iowa State University, 2310 Pammel Drive 339 Science Hall II, Ames, IA 50011, United States; Global Resource Systems, Iowa State University, Horticulture Hall, 2206 Osborn Dr Suite 105, Ames, IA 50011, United States

**Keywords:** Asiatic Wild Ass, endangered species, Iran, human-occupied landscape, long-distance movement, migration, nomadism, rangeland, wildland–human interface, کلمات کلیدی: ایران، تقابل انسان و حیات وحش، جابجایی های طولانی، گورخر آسیایی، چشم اندازهای انسانی ،کوچ، گونه های در معرض انقراض، مهاجرت، مرتع

## Abstract

Increasingly, the conservation of large and wide-ranging animals is challenged by environmental variability, static boundaries of protected areas, and the expansion of human activities. The Critically Endangered Onager (*Equus hemionus onager*) exemplifies these issues in Qatrouiyeh National Park (QNP) and the surrounding Bahram-e-Goor Protected Area (BPA) in Iran. Using GPS telemetry data from 9 adult females tracked over 2 years, we examined seasonal patterns in movement and incursions into cultivated lands in 2017 and 2018. Net squared displacement analyses indicated that most individuals exhibited range-resident behavior with occasional nomadic movements, with no evidence for migration (i.e., predictable movements to and from distinct seasonal ranges). Both monthly home range size and monthly movement rate varied seasonally, peaking in late spring and early summer (May–July). Individual home ranges were between 257 and 1,928 km^2^, while the extrapolated population-level home range (718 km^2^; 95% confidence interval = 276–1368) extended well outside QNP, covering large portions of the BPA. Occurrence distributions also expanded beyond the protected area into adjacent cultivated lands, highlighting the use of human-occupied areas by onagers. We recorded 2,285 (out of 72,168) GPS locations within cultivated lands and their surrounding 50 m buffer, with 60% of these incursions occurring immediately adjacent to QNP. Most incursions occurred at night and were strongly associated with both season and cumulative rainfall over the preceding 9 months. These findings emphasize how rainfall-driven variability in resource dynamics shapes the spatial distribution and behavior of onagers, thus elevating the risk of conflict with humans. The scale and seasonality of Onager movements highlight the need for flexible, landscape-level conservation strategies that extend beyond fixed park boundaries to encompass critical habitats and to mitigate conflict across the broader region.

## Teaser

Critically endangered onagers have large, seasonally shifting home ranges that extend beyond park and protected area borders. Rainfall over the previous 9 months drives incursions into surrounding cultivated lands, increasing the risk of human–wildlife conflict.

Wildlife conservation in multi-use landscapes poses challenges for conservation authorities, particularly where the resources required by wildlife intersect with subsistence livelihoods (Naughton-Treves et al. 2005; König et al. 2020). In particular, endangered species of large mammals often come into conflict with human activities by virtue of their extensive home ranges, low population densities, and often unpredictable seasonal movements (Woodroffe and Ginsberg 1998; Joly et al. 2019). Formally protected areas are limited in size and are fixed in location, thus constraining how individuals of species of conservation concern meet their resource requirements. Consequently, such individuals often are forced into human-dominated areas in times of resource scarcity, where they face heightened risks associated with human activities (Jones et al. 2018; Bowyer et al. 2019; Hoffmann 2022). Therefore, conservation strategies increasingly are implemented across entire landscapes to encompass a variety of land-use types and jurisdictions along wildland–urban interfaces (Bennett et al. 2015; Gigliotti et al. 2022; Maher et al. 2023).

Land sharing approaches can result in substantial costs to local communities, especially those reliant on small-scale farming or pastoralism (Oldekop et al. 2016; Schleicher 2018). Restrictions imposed on human activities (e.g., grazing, cultivation, fuelwood collection) can directly impact livelihoods by reducing food security and economic stability of local people, thereby affecting resource requirements of humans themselves (Cernea and Schmidt-Soltau 2006; West et al. 2006). Moreover, the presence of large herbivores or carnivores often leads to crop or livestock depredation, or even personal injury, compounding local hardship and generating resentment toward conservation efforts (Brockington and Igoe 2006; Dickman 2010). Without mechanisms for compensation, benefit-sharing, or inclusive governance, efforts to conserve wildlife are likely to be perceived as externally imposed, undermining both their social legitimacy and long-term viability.

In arid and semi-arid environments, humans and wildlife must cope with unpredictable environmental conditions, particularly in relation to rainfall (Reynolds et al. 2007). In these ecosystems, rainfall drives forage availability—influencing both plant biomass and nutritional quality—which in turn shapes movements of wildlife and affects the livelihoods of pastoralists and rural communities (Mueller et al. 2011; Boone et al. 2018; Esmaeili et al. 2021). On the one hand, during dry periods, the scarcity of forage often forces wild ungulates to expand their movements across larger areas in search of ephemeral resources (Fryxell and Sinclair 1988; Sullivan and Rohde 2002). This pattern not only imposes energetic costs, but also brings wild ungulates into closer proximity to human settlements, cultivated lands, and livestock (Owen-Smith et al. 2010; Suryawanshi et al. 2013; Abrahms 2021). On the other hand, during wet periods rainfall-driven vegetation growth temporarily increases the forage available for both wild ungulates and livestock, potentially alleviating competition (Odadi et al. 2011; Valls-Fox et al. 2018; Esmaeili et al. 2024). However, wet periods also encourage agricultural expansion and intensification of grazing pressure, as pastoralists and rural communities take advantage of improved but temporary conditions (Homewood et al. 2009; Derry and Boone 2010). This broadening of the wildland-human interface can create ecological traps as conditions dry, when forage availability collapses and both wildlife and humans concentrate on dwindling resources (Derry and Boone 2010; Reid et al. 2014). As a result, the potential for human–wildlife conflicts intensifies leading to increased crop raiding, competition with domestic stock, and retaliatory killings (Abrahms 2021). For species already adapted to marginal environments, this combination of environmental variability and anthropogenic pressures can compromise population viability (Fynn and Bonyongo 2011; Nandintsetseg et al. 2019). The intersection of environmental variability and human land use thus represents a major challenge for the conservation and management of arid-zone ungulates, requiring flexible, landscape-level strategies that account for both climatic fluctuations and socio-economic realities (Reid et al. 2014).

The Asiatic Wild Ass (*Equus hemionus*) exemplifies how the conservation of a wide-ranging wild ungulate encompasses a variety of land uses in a resource-limited environment (Kaczensky et al. 2008, 2025b). Across much of its current geographic range, the Asiatic Wild Ass increasingly inhabits landscapes undergoing agricultural expansion and intensified pastoralism (Nandintsetseg et al. 2016; Xu et al. 2022; Kaczensky et al. 2025b) while maintaining a wide-ranging, nomadic lifestyle that enables it to exploit highly variable patterns of rainfall and forage availability (Kaczensky et al. 2008; Nandintsetseg et al. 2016). In the Gobi Desert, Asiatic Wild Ass track patchy precipitation events, enabling them to use transient flushes of vegetation across a low productivity landscape (Nandintsetseg et al. 2016). This nomadism increases their exposure to anthropogenic threats, as movements frequently extend beyond the boundaries of formally protected areas into landscapes dominated by pastoralism and impacted by linear human infrastructure (Kaczensky et al. 2011). In Iran, the only remaining population of the Critically Endangered Onager subspecies (*E. h. onager*) is confined to the Bahram-e-Goor Protected Area (BPA) and its core zone of Qatrouyeh National Park (QNP; Kaczensky et al. 2025a). Here, a formerly small and restricted Onager population has grown to a size of ca. 1,300 individuals, such that intensive management interventions—the exclusion of human activities within QNP, supplemental water and forage provisioning, and strict patrolling—now are required to ameliorate conflicts between humans, their livestock, and onagers (Esmaeili et al. 2024). Despite their current protection, onagers regularly encounter risks associated with human activities including retaliatory killing, collisions with vehicles, and competition with domestic livestock (Esmaeili et al. 2019, 2024; Kaczensky et al. 2025a). The challenge of conserving onagers, therefore, mirrors global challenges faced by wide-ranging and nomadic ungulates: protected areas that are too small, with boundaries that are misaligned with the dynamic resource requirements of wide-ranging species. While conservation interventions helped maintain the current population within the QNP–BPA complex, they ultimately are short-term solutions that do not address the underlying drivers of human–wildlife conflict.

Historical and anecdotal reports suggest that part of the Onager population in the QNP–BPA complex regularly undertakes long-distance movements. Onagers leave QNP in mid- to late spring to forage in cultivated lands within and adjacent to the BPA, then return in the fall when seasonal rains trigger the growth of fresh annual grasses (Jowkar and Nabian 2005). Local farmers report that Onager incursions into cultivated lands peak during the summer months (June–September; Esmaeili et al. 2019), which are perceived to be driven by forage shortages within QNP and the subsequent seasonal movement of onagers. To explore and quantify these behaviors, we aimed to address: (1) whether and to what extent onagers expand their movements seasonally; and (2) the timing and frequency of their incursions into cultivated lands. Drawing on knowledge typical of other Asiatic Wild Ass populations (Nandintsetseg et al. 2016), we hypothesized that: (1) onagers do not follow predictable long-distance movements, but instead move opportunistically in response to changing resources (i.e., onagers are nomadic); (2) incursions into cultivated lands are driven primarily by forage depletion within QNP, rather than following a consistent seasonal pattern; and (3) while onagers require vast areas, their space use fluctuates in ways that could inform targeted, seasonal strategies to better conserve habitat and safeguard the population.

## Methods

### Study area

**Fig. 1:**
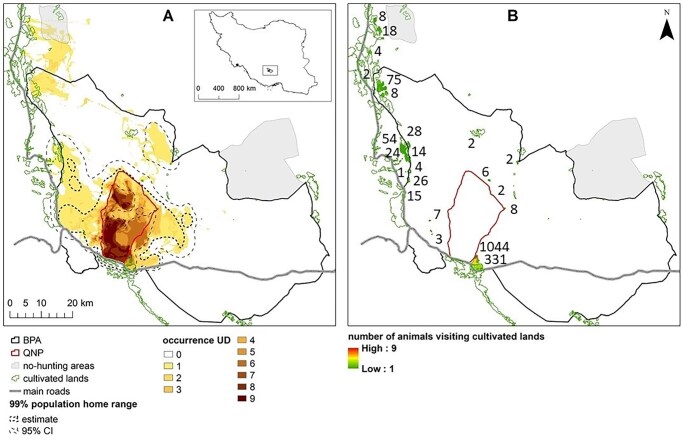
Location of Bahram-e-Goor Protected Area (BPA) and Qatrouiyeh National Park (QNP) in south-central Iran (inset). (A) The spatial distribution of 9 GPS-collared onagers in 2017–2018 is represented by their occurrence utilization distribution (UD), where values range from 0 (no individuals) to 9 (used by all collared individuals). The 9 collared onagers primarily remained within QNP and its surrounding area, although some individuals moved beyond the BPA into the adjacent no-hunting area. The estimated 99% population-level home range using auto-correlated density estimator is shown with a black dotted line, and the 95% lower and upper confidence intervals are shown in gray dotted lines. (B) Cultivated lands intersecting the 99% UD for 1 to 9 onagers are highlighted with a solid color, proportional to the number of animals visiting each farm. Cultivated lands in which incursions did not occur are depicted as outlines. Numbers on the map indicate the count of Onager GPS locations recorded within each cultivated field and its 50 m buffer between 2017 and 2018.

In 2017 and 2018, we conducted our study within the Bahram-e-Goor Protected Area (BPA, established in 1972, covering an area of 3,747 km^2^), encompassing the Qatrouiyeh National Park (QNP, established in 2008, with an area of 310 km^2^; Fig. 1). The region experiences an arid, seasonal climate characterized by a mean annual temperature of 21.27 °C ± 0.29 SE and mean annual rainfall of 185.00 mm ± 107.00 (total annual rainfall in 2017 and 2018 were 290 and 78 mm, representing a wet and a dry year, respectively). The driest months are from May to September (with an average 0.00 mm ± 0.00 SE rainfall), while February is the wettest month with 93.50 mm ± 76.00 of rainfall, based on climate data from 2017 and 2018 (Iran Meteorological Organization, http://www.irimo.ir). Vegetation cover is sparse and is predominantly composed of *Artemisia sieberi*. Other species of wild ungulates in our study area include Jebeer Gazelle (*Gazella bennettii*), Mouflon (*Ovis gmelini*), Wild Goat (*Capra aegagrus*), and Wild Boar (*Sus scrofa*). Large carnivores such as Gray Wolf (*Canis lupus*) and Striped Hyena (*Hyaena hyaena*) inhabit the area; however, no instances of predation by these species on adult onagers have been recorded.

QNP was designed to safeguard the core habitats of onagers by prohibiting human and livestock access, establishing permanent water sources, fencing the southern border of the park along the main road, and implementing strict antipoaching patrols. In 2017 and 2018, the Iranian Department of Environment documented a population of 793 ± 62 SE onagers in QNP and BPA, with the majority of individuals concentrated within QNP. As of 2024, an estimated population of approximately 1,358 individuals has been reported based on attempted total counts (Hemami, M. R. Iranian Department of Environment, written comm. 2025).

In contrast to QNP, BPA is inhabited by approximately 4,000 seminomadic pastoralists and small-scale farmers who primarily raise sheep (*Ovis aries*) and goats (*Capra hircus*), with an average density of about 14 head of sheep and goat (2.1 Livestock Units) per square kilometer (Hemami, M. R. Iranian Department of Environment, written comm. 2017). Cultivation is concentrated mainly around villages within and along the border of BPA. The primary crops include wheat (*Triticum spp*.), Barley (*Hordeum vulgare*), Alfalfa (*Medicago sativa*), Corn (*Zea mays*), and fruit trees such as Pistachio (*Pistacia vera*) and Pomegranate (*Punica granatum*; Esmaeili et al. 2019). Occasionally, cultivated lands within and adjacent to BPA face seasonal incursions by onagers, a trend that has been on the rise due to the increasing Onager population (Esmaeili et al. 2019). The crops most vulnerable to depredation include Alfalfa, Wheat, Barley, fruit trees, vegetables, and Corn (Esmaeili et al. 2019).

### Onager GPS telemetry

Between December 2016 and January 2017, 9 female onagers were captured in QNP using corral traps, then fitted with GPS telemetry collars (Vertex Lite 2 Iridium, Vectronic Aerospace GmbH, Berlin, Germany; refer to Esmaeili et al. (2024) for capture and deployment protocols). We collared females exclusively because they are consistently integrated into social groups (as opposed to males, for which group membership is more fluid) and thus provide a more reliable representation of group-level space use, while also reducing the risks of collar loss and animal-welfare concerns associated with stallions (Jung et al. 2018; Schoenecker et al. 2024). Collars were programmed to acquire GPS fixes every 2 hours and detach automatically after 2 years. To reduce potential biases caused by postcapture effects, we excluded GPS data from the first 2 weeks following collaring (Dechen Quinn et al. 2012). Additionally, we filtered out locations with dilution of precision values greater than 10 (which comprised 0.04% of the locations) to ensure spatial accuracy (Lewis et al. 2007). After filtering, the dataset included 72,168 GPS locations collected from January 2017 through December 2018. The mean and SD of number of GPS locations per 24 hrs per individuals was 11.96 ± 0.03. All capture, marking, and tracking procedures complied with ethical guidelines and were approved by the Iranian Department of Environment (No. 95/12631) and the University of Wyoming Institutional Animal Care and Use Committee (No. 20160225SE00212-01), following standards of the American Society of Mammalogists (Sikes et al. 2016). To verify the independence of movements of the collared animals, we calculated coefficients of association (CA) for all dyads using the “wildlifeDI” package in R (Long et al. 2022). The CA measures the proportion of simultaneous GPS locations (within 2 hours) occurring within 200 m. Values above 0.5 typically indicate attraction, while values below 0.5 suggest no attraction. In our study, CA values were consistently low (mean ± SD = 0.04 ± 0.05; range = 0.00–0.19), indicating spatial independence among all individuals.

### Movement and space use patterns

Across both study years, we investigated movement strategies of individuals by analyzing and interpreting net squared displacement (NSD) profiles over time. NSD is defined as the squared Euclidean distance between initial location of an animal and each subsequent position along its movement trajectory (Turchin 1998; Bunnefeld et al. 2011). The temporal structure of NSD curves reflects distinct movement strategies at broad spatial and temporal scales including migration, dispersal, residency, and nomadism (Cagnacci et al. 2011; Singh et al. 2016). While visual interpretation of NSD plots can reveal general movement patterns, the use of model-based classification greatly improves consistency and inferential power, especially for distinguishing between complex or irregular movement behaviors (Bastille-Rousseau et al. 2016). To classify Onager movement strategies, we applied the hierarchical modeling framework proposed by Bastille-Rousseau et al. (2016) using the R package “Ismnsd”. This approach uses latent-state models to identify shifts in large-scale movements by evaluating the distribution and temporal dynamics of NSD values. For example, low-variance NSD profiles are typically associated with residency, while gradually increasing or more uniform NSD curves may indicate nomadism or dispersal. To minimize noise from short-term exploratory movements and GPS error, we averaged location of each individual within 24 hr periods prior to analysis (Bastille-Rousseau et al. 2016). In instances where automated model selection failed to converge on a specific strategy (likely due to limited displacement or irregular movements), we visually interpreted the NSD trajectories to assign the most appropriate classification, as recommended in previous studies (Cagnacci et al. 2011; Spitz et al. 2017).

To investigate seasonal shifts in space use and movement rate of onagers, we calculated 2 metrics for each collared individual during each study month (monthly home range size and average monthly movement rate) as follows. First, we applied variogram analysis using the “ctmm” package in R (Calabrese et al. 2016) to identify months in which individuals did not exhibit range-resident behavior. Variograms measure the semivariance in position as a function of the time lag between relocations, and thus are useful for interpreting space-use dynamics (Fleming et al. 2014). Specifically, the shape of the variogram can indicate specific movement modes: an asymptote suggests stable home range behavior, while a continuously increasing variogram reflects nonresidency or transient movement. Monthly home ranges were estimated using the autocorrelated kernel density estimator (AKDE) for individuals identified as range residents in a given month (Fleming and Calabrese 2023). Before home range estimation, we fit the best-supported continuous-time movement model to monthly tracking data for each individual using the perturbative hybrid restricted maximum likelihood method (Silva et al. 2022). We excluded monthly home ranges with an effective sample size < 6, as these are considered unreliable (Fleming et al. 2019; Silva et al. 2022; Barbour et al. 2025). The effective sample size was automatically estimated by the “ctmm” package as the duration of the track divided by the home range crossing time (Silva et al. 2022). We calculated monthly speed as the mean speed of the Gaussian movement process, using the speed function in the “ctmm” package (Noonan et al. 2019).

We estimated annual home ranges at individual and population levels using the AKDE method. For individuals not exhibiting range residency, we excluded locations associated with long-distance exploratory movements based on visual inspection of movement tracks (Silva et al. 2022). We calculated 99% AKDE volume contours to represent individual home ranges. Due to the conservation implications of our work and our relatively small sample size, we used the 99% AKDE contour instead of the conventional 95% to capture a more comprehensive representation of space use, encompassing most areas used by the collared individuals. We used the pkde function in “ctmm” to extrapolate the home range at the population level and used the meta function in “ctmm” to estimate the average home range size for the population (Fleming et al. 2022; Fleming and Calabrese 2023). This function applies a χ^2^–Inverse Gamma meta-analytical approach, which is more robust than a simple arithmetic mean. By incorporating individual uncertainty, the method assigns lower weights to less certain estimates, reducing bias due to variation in effective sample sizes (Fleming et al. 2022).

### Incursions into cultivated land

To evaluate the extent to which cultivated lands were used by onagers, we analyzed the occurrence distributions of the collared individuals. We estimated the occurrence distributions using a generalized time-series Kriging framework incorporating an integrated Ornstein–Uhlenbeck (OU) movement model in the “ctmm” package (Calabrese et al. 2016; Fleming et al. 2016). This method is suitable for modeling non–resident behaviors because it does not rely on the assumption of a stable home range and instead characterizes where an animal was likely to be during a specified time (Noonan et al. 2021). We derived 99% occurrence distributions with 95% confidence intervals for each individual across the study area to conservatively represent their space use during our study period. To identify areas in which Onager activity was concentrated, we converted the occurrence distribution of each individual into a binary presence/absence raster. These rasters were then overlaid to produce a cumulative occurrence map, where pixel values ranged from 1 (used by a single individual) to 9 (used by all individuals). Finally, we intersected this cumulative raster with geospatial data on cultivated lands to identify agricultural areas most frequently visited by onagers during the study period using packages “terra” (Hijmans et al. 2022) and “sf” (Pebesma 2018).

We investigated the factors influencing Onager incursions into cultivated lands by integrating a remotely sensed vegetation index and time series of rainfall data. To capture variation at both fine and broader temporal levels, we analyzed data at 2 scales: 8 d intervals (matching the temporal resolution of the vegetation index) and 1 mo intervals. Given the aridity of the study area and the relatively low variability in vegetation and rainfall, we considered these 2 scales complementary. We used the Modified Soil-Adjusted Vegetation Index (MSAVI; Qi et al. 1994) derived from surface reflectance captured by the Moderate Resolution Imaging Spectroradiometer (MODIS) Terra satellite (Version 6.0 MOD09Q1), with a spatial resolution of 250 × 250 m and a temporal resolution of 8 d to quantify vegetation greenness. In sparsely vegetated and mineral-rich regions, MSAVI can more accurately capture vegetation dynamics than nonadjusted indices such as the Normalized Difference Vegetation Index (Qi et al. 1994). Additionally, we incorporated daily rainfall data (in mm) recorded at a rain station adjacent to QNP (station ID: 171416, Latitude: 29.148, Longitude: 54.7; Iran Meteorological Organization, http://www.irimo.ir), to evaluate the role of rainfall in shaping patterns of cultivated land incursions. We defined an incursion as the presence of Onager GPS locations within cultivated lands and a 50 m buffer around them. We selected a 50 m buffer based on the small size of cultivated lands in the area (Fig. 1) and on personal observations, as well as reports from local residents and rangers, to avoid conflating GPS fixes from animals that move in close proximity to cultivated lands with those actually entering fields. In addition, because some farmers use temporary fencing or seasonal protection (e.g., security personnel who keep watch at night), we selected a 50 m buffer to exclude points where animals approached but did not use cultivated land. Incursions were counted for every 8 d interval and for every monthly interval. To characterize vegetation at incursion sites, we extracted MSAVI values corresponding to each Onager location using the nearest available MSAVI layer in time. For each time interval, we averaged the MSAVI values of all Onager locations in cultivated lands to represent greenness during the incursion (i.e., cultivated land MSAVI). To characterize conditions during periods when no incursion occurred, we randomly sampled 25 (for 8 d intervals) and 60 (for monthly intervals) points within cultivated lands—reflecting the average number of incursions per period across the study—and assigned a random date within the corresponding interval. We then extracted and averaged MSAVI values from those points to represent cultivated lands MSAVI during nonincursion periods. To assess vegetation conditions in the core habitat of onagers (within QNP) at the time of incursions, we randomly sampled an equal number of GPS locations from QNP for each event and extracted corresponding MSAVI values, then averaged the values across the associated interval (i.e., QNP MSAVI). By subtracting QNP MSAVI from the cultivated lands MSAVI, we derived a vegetation contrast index representing the difference in greenness between the 2 areas (i.e., ΔMSAVI). We hypothesized that onagers would be more likely to move onto cultivated lands when vegetation within the park was limited, and subsidized forage remained available in cultivated lands. To account for the effects of rainfall on cultivated land incursions, and the delayed response of vegetation to rainfall in arid environments, we aggregated daily rainfall totals over a range of lag periods. In arid environments, rainfall is often infrequent and highly variable, and its effects on vegetation growth can manifest over extended time frames depending on soil moisture, plant functional types, and rainfall intensity and duration (Huxman et al. 2004; D’Odorico et al. 2013). By testing multiple lag periods, we aimed to capture both short-term and cumulative rainfall effects on Onager forage. This approach incorporates the fact that vegetative responses to rainfall in water-limited systems are not always immediate and depend on how rainfall events interact with phenology, soil water retention, and previous moisture conditions (Sala et al. 2012). At the 8 d scale, we summed rainfall over the prior 7, 15, and 21 days; and 1, 2, 3, 4, 5, 6, 9, and 12 mo. At the monthly scale, we used rainfall from the current month and lagged totals from the preceding 2, 3, 4, 5, 6, 9, and 12 mo. All GIS and statistical analyses were performed in the R environment (R Core Team 2013).

### Statistical analyses

To assess seasonal variation in Onager movements, we fit generalized linear mixed models (GLMMs) using the glmer function from the “lme4” package (Bates et al. 2015) to monthly movement rates (km/day) and range sizes (km^2^). Because our response variables were continuous and strictly positive measures that exhibited right-skewed distributions, we used a Gamma distribution with a log link function in our models. The month was modeled as a cyclical variable using sine and cosine transformations to capture seasonal patterns. To account for individual variation, we included a random intercept for animal ID in our models.

To evaluate the effects of rainfall and seasonality on the number of incursions per 8 d (*n *= 92 8 d periods), we fitted a zero-inflated negative binomial (ZINB) regression model using the “glmmTMB” package (Brooks et al. 2017). Prior to model fitting, we examined the correlation between MSAVI values and the cumulative rainfall at different lags using Pearson’s correlation coefficients. We did not include variables with high correlation (*r *> 0.5) in the same model. We constructed a series of candidate models with QNP MSAVI, ΔMSAVI, cumulative rainfall at different lags, study year, and the day of year (modeled as a cyclic variable using sine and cosine transformations). We compared the candidate models using the Akaike Information Criterion (AIC; Akaike 1974) and selected the most parsimonious model (i.e., that with the lowest AIC value). We screened candidate models for uninformative parameters (Arnold 2010; Leroux 2019). Predictors that failed to improve log-likelihood while increasing model complexity were considered uninformative and not interpreted (Arnold 2010). All predictor variables were scaled prior to model fitting to improve convergence and comparability of effect sizes.

At the monthly scale, we limited each candidate model to a single predictor to avoid overfitting, due to the small sample size (*n *= 24 months) and high collinearity among MSAVI and rainfall variables (as indicated by Pearson’s correlation coefficients). We constructed a series of candidate models to assess the relationship between environmental covariates and the number of monthly farm incursions including models with QNP MSAVI, ΔMSAVI, cumulative rainfall at different lags, study year, and month (modeled as a cyclic variable using sine and cosine transformations). We fitted generalized linear models (GLMs) with a negative binomial distribution and log link function using the “MASS” package (Ripley et al. 2013). Models were compared using AIC to identify the variables that best predicted Onager incursion frequency at the monthly scale.

## Results

### Movement and space use patterns

Our NSD analysis classified 14 out of 18 animal-year Onager movement trajectories as residents and 1 as nomadic, and was unable to classify 3 animal-years (which were flagged as “inspection needed”; Supplementary Data SD1). Visual interpretation of NSD plots revealed no clear patterns of recurring seasonal movements (Supplementary Data SD1), suggesting that onagers in the study area do not exhibit repeatable long-distance movements. For the 2 animal-years not classified as residents, visual inspection of the NSD plots showed irregular movement patterns consistent with nomadic behavior (IDs: 21720 in 2017; 22116 in 2017 and 2018; and 22117 in 2018). Variogram inspection of individuals 21720 and 22116 indicated nonresident behavior during certain months, but not throughout the entire year. These individuals appeared to traverse broader areas than others, exhibiting nomadic movements in some months. Two animals (IDs 22117 and 22116) initiated long-distance movements directed outside the BPA in April 2018. The 2 animals traveled together 114 km over 4 days, moving from cultivated lands adjacent to the park to areas in the northwest outside the BPA including a designated no-hunting area where hunting had been prohibited for 10 years (Fig. 1). The collar of one animal dropped prematurely and the fate of the animal is unknown, whereas the other animal was later found dead; both events occurred outside the BPA boundary. The cause of the premature collar release and the mortality event could not be determined. We removed locations associated with the movement of these 2 individuals after 20 April 2018 in our home range estimations because those locations did not represent the long-term home range of animals, but instead seemed to be long-distance excursions (Fieberg and Börger 2012).

**Fig. 2:**
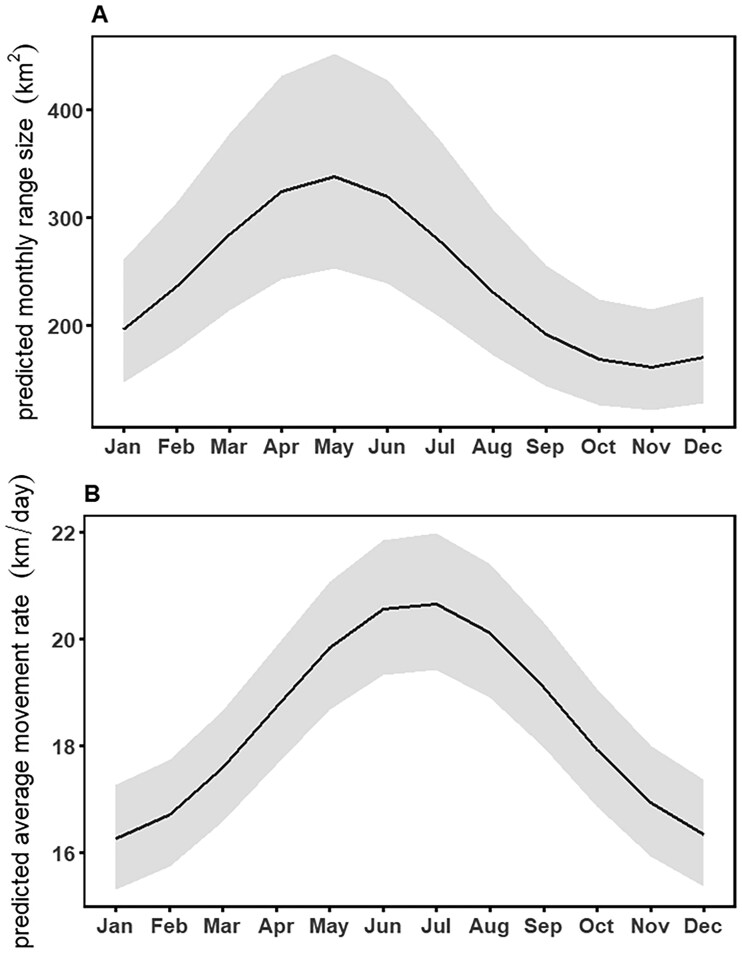
Estimated monthly (A) range size and (B) average movement rate (solid black lines), with 95% confidence intervals (gray bands), as functions of time for nine GPS-collared onagers in south-central Iran during 2017 and 2018. We modeled monthly range size (*n *= 183 animal-months) and average movement rate (*n *= 195 animal-months) using generalized linear mixed models, with the month as a cyclical fixed effect (via sine and cosine transformations). We included animal ID as a random intercept to account for variability across individuals.

At the monthly scale, we excluded movement models that did not reflect range residency, had small effective sample sizes, or produced implausibly large monthly home ranges (*n *= 17 animal-months; Supplementary Data SD2). For the speed analyses, estimates were not available when the best-fitting model was an OU process (*n *= 5 animal-months; Supplementary Data SD2), because the OU model does not incorporate velocity autocorrelation (Calabrese et al. 2016). Our GLMM models revealed significant seasonal variation in both monthly home range size and movement rate (Fig. 2). Monthly home range sizes of individuals increased during certain periods of the year (sine of month: *β*  =  0.20 ± 0.08 SE, *P* = 0.016) and decreased during others (cosine of month: *β* = –0.31 ± 0.08, *P* < 0.001), indicating a strong cyclical pattern (Fig. 2A). Home ranges were smaller in September–January and larger in spring and early summer (April–June) with a peak in May, in which they exceeded 300 km^2^ (Fig. 2A). Monthly movement rates also varied seasonally (Fig. 2B), decreasing during some parts of the year (cosine of month: *β* = –0.11 ± 0.02 SE, *P* < 0.001), with a marginal effect of sine of the month (*β* = –0.04 ± 0.02 SE, *P* = 0.050). Predicted average movement rates increased from January through June, peaked in June–July at approximately 21 km/day, and declined steadily through December, reflecting a clear seasonal expansion and contraction in space use of individuals (Fig. 2B).

The estimated 99% of home range size on the 9 collared onagers ranged between 257 and 1928 km^2^ (Supplementary Data SD3 and SD4), with the estimated average population-level home range size of 567 km^2^ (95% CI = 366.42–838.30). The extrapolated population-level 99% AKDE was 718.30 km^2^ (95% CI = 276.07–1368.32) and encompassed the QNP and approximately 383 km^2^ of the BPA (Fig. 1).

### Incursions into cultivated land

We recorded 2,285 (3.2%) of the GPS locations within cultivated lands and their 50 m buffers including 361 (0.5%) locations in 2017 and 1,924 (2.7%) in 2018 (Fig. 1; Supplementary Data SD5). Notably, 98% of these incidents occurred during nighttime hours. All 9 collared onagers visited cultivated fields adjacent to the QNP, which accounted for 60% of all recorded incursions (Fig. 1). Another hotspot was located in the western and northwestern borders of the BPA, where at least 2 animals visited cultivated lands. Onagers were reported to forage on crops and drink water when observed on cultivated lands.

**Fig. 3:**
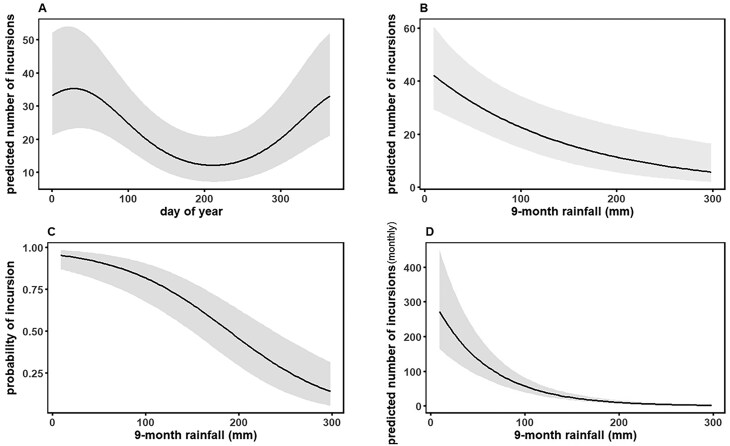
Predicted number of Onager incursions into cultivated lands as a function of season and rainfall in south-central Iran in 2017 and 2018. Estimated number of incursions per 8-day period as a function of day of year (A) and cumulative rainfall over the prior 9 months (9-month rainfall, B) based on a zero-inflated negative binomial regression model. The predicted probability of incursion (zero-inflation component) increases with 9-month rainfall, indicating a reduced likelihood of incursion under wetter conditions (C). The monthly number of Onager incursions decreases with increasing 9-month rainfall, as predicted by a generalized linear model (D). Solid lines represent model estimates and gray bands are 95% confidence intervals.

The number of cultivated land incursions every 8 days by onagers was best described by both seasonality and the previous 9 mo cumulative rainfall (Supplementary Data SD6). The final model included 9 mo cumulative rainfall as a smoothing (using function s from package “mgcv”; Wood and Wood 2015) term to capture potential nonlinear effects. Seasonality was modeled using cyclic sine and cosine transformations of the Julian day to reflect intra-annual variation. A zero-inflation component was included to account for excess zeros in the response, modeled as a function of the cumulative 9 mo rainfall. The negative binomial family was used to accommodate overdispersion in the response variable. At the 8 d scale, we found no significant correlation between park greenness and rainfall at any time lag, likely due to sparse vegetation and low rainfall (Supplementary Data SD7). At this resolution, MSAVI values in the park and ΔMSAVI were not correlated (*r *= 0.02, *P *= 0.93), while ΔMSAVI was strongly correlated with cultivated land MSAVI (*r *= 0.98, *P *< 0.001). A positive relationship between rainfall and greenness emerged after a 3 mo lag, peaking at 6 to 9 mo lag (Supplementary Data SD7). Incursions peaked seasonally, as indicated by the significant effect of the cosine component of the day of the year (*β*  =  0.47 ± 0.18 SE, *P* = 0.008), while the sine component was marginally significant (*β*  =  0.25 ± 0.15, *P* = 0.085; Fig. 3A). The cumulative rainfall over the previous 9 mos had a nonlinear and negative effect on incursion rates, with peak incursion activity occurring at moderate to low rainfall levels (*β* = –0.79 ± 0.22 SE, *P* < 0.001), suggesting that incursions increased during prolonged dry periods (Fig. 3B). The zero-inflation component further supported this relationship, showing that the probability of observing 0 incursions was significantly higher when cumulative rainfall over the previous 9 mos was high (*β*  =  1.95 ± 0.35 SE, *P* < 0.001). From the ZINB model, rainfall significantly influenced both the frequency and the possibility of cultivated land incursions by Onager (Fig. 3C).

At the scale of months, MSAVI values in the QNP and cultivated areas were significantly correlated (*r *= 0.59, *P *< 0.001; Supplementary Data SD8). The strongest correlations between greenness and rainfall were observed at a 9 mo lag in both the QNP and cultivated areas (*r *= 0.78 and *r *= 0.80, respectively; *P *< 0.001; Supplementary Data SD8). The number of cultivated land incursions per month was strongly and negatively associated with cumulative rainfall over the previous 9 mos (Supplementary Data SD9; Fig. 3D). Incursion frequency declined significantly with increasing rainfall (*β* = –2.05 ± 0.21 SE, *P* < 0.001), indicating that onagers were more likely to invade cultivated areas during prolonged dry periods (Fig. 3D).

## Discussion

Our study is the first to provide GPS-telemetry-based information on the movement ecology and human–wildlife interactions of critically endangered onagers inhabiting an arid, multi-use landscape. By combining GPS tracking with remotely sensed environmental data, we found that onagers within the QNP–BPA complex exhibited a combination of range-residency and nomadic movement strategies, with substantial seasonal variation in both home range sizes and movement rates. Four of the 9 collared animals displayed nomadic behavior during certain periods and roamed beyond the boundaries of the protected area. Moreover, the average population-level home range was nearly twice the size of QNP, which reflects global conservation challenges seen in wide-ranging species, where fixed protected areas often fail to accommodate the full range of the target species (Runge et al. 2014). Additionally, 2 individuals traveled more than 110 km within 4 days, demonstrating their ability to cross large landscapes in short timeframes. These observations highlight the critical role of landscape connectivity in effective conservation planning within fragmented environments (Rezvani et al. 2024). One of these animals was later found dead outside protected boundaries, while the fate of the other remains unknown. These cases expose critical conservation gaps and highlight the heightened vulnerability of individuals in areas with limited or inconsistent protection. Meanwhile, incursions into cultivated lands were closely linked to prolonged drought conditions, reflecting how rainfall-driven shifts in vegetation influence movement patterns and amplify the risk of human–wildlife conflict in arid environments.

Although historical accounts and anecdotes have suggested regular long-distance movements (i.e., migrations; Jowkar and Nabian 2005), onagers in our study exhibited a combination of range residency and nomadism, with most individuals maintaining stable home ranges throughout the year. Previous studies and local reports in the area were based on direct observations of animals roaming outside the park and protected area annually. While these accounts may be true, they are often inaccurate due to the lack of individual identification and the conflation of nomadic long-distance movements with true migration. These findings highlight the need for GPS telemetry of a larger number of individuals over a longer period to better understand the dynamics of population-level space use. Additionally, movement rates and monthly ranges both expanded substantially during late spring and summer, coinciding with the peak of the dry season. These seasonal expansions suggest that while onagers do not engage in predictable long-distance movements, they adjust their movements in response to seasonal resource scarcity, consistent with nomadism (Kaczensky et al. 2008; Mueller and Fagan 2008). The timing of home range and movement expansions did not fully coincide with the peak periods of cultivated land incursions, implying that factors beyond simple forage depletion—for example, events like parturition and mating, which occur in late April to early June (Jowkar and Nabian 2005)—may also influence seasonal movements. The absence of strong, recurring large-scale movements and the predominance of resident strategies likely reflect a combination of ecological factors (e.g., patchy, unpredictable rainfall) and anthropogenic pressures (e.g., livestock competition, human disturbance) shaping Onager space use in this arid, multi-use landscape (Esmaeili et al. 2024).

Faced with shifting and unreliable resources, many animals in arid and semiarid regions adopt nomadic movement strategies to survive (Mueller et al. 2011). In these systems, forage and water fluctuate dramatically across space and time, favoring opportunistic, resource-driven movements. In contrast to migratory species, which undergo predictable long-distance movements in space and time (Kauffman et al. 2021), nomadic species move flexibly and unpredictably across landscapes, tracking ephemeral patches of forage and water rather than adhering to consistent patterns (Dingle and Drake 2007; Mueller and Fagan 2008). While such ecological flexibility enables persistence in stochastic environments, it also presents challenges for conservation. Because the spatial distribution of key resources shifts from year to year and even season to season, it becomes difficult to consistently identify and protect critical habitats (Boone et al. 2018; Kauffman et al. 2021).

In recent years, managers have increasingly recognized the need for protected area strategies that are dynamic: systems that adjust spatially or temporally in response to shifting environmental conditions (Pressey et al. 2007; Runge et al. 2014; Allen and Singh 2016). Such flexible approaches aim to better align wildlife conservation with the dynamic requirements of species for resources that themselves vary spatially and temporally. In and along the wildland–human interface where wildlife and humans share limited resources, providing targeted, seasonal protection may represent a practical and cost-effective strategy. This is particularly the case for countries in which wildlife authorities must work with very limited budgets. For nomadic or highly mobile species, dynamic protected areas offer a promising framework by incorporating environmental unpredictability, seasonal movements, and anthropogenic pressures into conservation planning (Reynolds et al. 2007). Our results showed that onagers expand their ranges and increase their movement rates during dry seasons, and thus are more likely to come into conflict with humans during prolonged droughts. Ideally, the cultivated fields within the BPA would be relocated and compensated with land outside the BPA or farther from QNP; however, this does not appear feasible or achievable in the short term. The population-level home range estimates that we provided could serve as a baseline for strategically expanding conservation interventions such as intensified seasonal patrolling during the critical spring and summer periods when onagers exhibit the largest home range sizes. The population-level home range, the no-hunting area in the northwest of the BPA, and the intervening movement corridor could be targeted as potential sites for long-term temporal or permanent expansion of QNP or formal recognition as buffer zones to safeguard Onager habitat year-round.

In addition to expanding protection seasonally, static strategies including preventing the spread of cultivation, assisting locals with fencing their fields, offering compensation for incursion-prone fields to be relocated outside the protected area or left fallow, and encouraging a shift from highly attractive crops (i.e., Alfalfa, Wheat, and Barley; Esmaeili et al. 2019) to less accessible alternatives such as trees (depending on water availability), could help sustain both local livelihoods and Onager habitats. Moreover, the strong predictive relationship that we observed between cumulative 9 mo rainfall and Onager incursion rates presents an opportunity to develop early warning systems that protect local cultivations. By integrating rainfall forecasts into seasonal patrolling schedules, identifying conflict hotspots, and activating community alert systems, managers could reduce crop losses and prevent resentment toward onagers and their conservation. Such resentment often stems from the perception among local communities that wildlife protection is prioritized over human well-being (Bennett et al. 2017; Esmaeili, S. oral comm., with locals 2016). However, implementing both dynamic and static protection strategies simultaneously remains challenging and requires adaptive management along with strong, sustained cooperation with local communities.

Conserving nomadic species such as the Asiatic Wild Ass in human-modified landscapes requires management strategies that are dynamic and flexible to shifting environmental conditions. Static protected areas, while important, typically are insufficient to conserve wide-ranging mammals and would need to be combined with seasonal expansion of protection when static approaches are not successful on their own. Mobile conservation units that could provide seasonal patrolling, periodic compensation for leaving lands uncultivated, and co-management agreements with local communities may offer more straightforward, responsive, and equitable mechanisms for mitigating human–wildlife conflicts in this landscape. Over ­decades to centuries, conservation success will likely depend on ­integrated landscape-scale approaches that recognize the dynamics of resource availability, promote coexistence between wildlife and ­people, and address the underlying socio-economic drivers of human-wildlife conflicts.

## Supplementary Material

gyag017_Supplementary_Data

## Data Availability

Data available upon request.

## Supplementary data

**Supplementary Data SD1**. Plots of net squared displacement.

**Supplementary Data SD2**. Monthly movement-model summaries.

**Supplementary Data SD3**. Individual and population home range size estimates.

**Supplementary Data SD4**. Movement model summaries.

**Supplementary Data SD5**. Number of GPS locations recorded in cultivated lands.

**Supplementary Data SD6**. Model selection results for zero-inflated regression models.

**Supplementary Data SD7**. Pearson correlation matrix 8-day intervals.

**Supplementary Data SD8**. Pearson correlation matrix monthly intervals.

**Supplementary Data SD9**. Model selection results for generalized linear models.
