# Supplementary material for: When the desert dries: rainfall drives conflicts and conservation challenges for onager (Equus hemionus onager)
Source: J Mammal. 2026 May 1;107(3):462–73. doi: 10.1093/jmammal/gyag017 (PMC13271766; doi:10.1093/jmammal/gyag017)
Supplement: gyag017_Supplementary_Data [file gyag017_supplementary_data.zip › Esmaeili_et al_S_materials_24Oct2025.docx]

**Supplementary Data**

**When the Desert Dries: Rainfall Drives Conflicts and Conservation Challenges for a Critically Endangered Equid**

Saeideh Esmaeili^1 *^, Kathryn A. Schoenecker^2,3^, Mahmoud-Reza Hemami^4^, Petra Kaczensky^5,6,7^, Chris Walzer^6,8^, and Jacob R. Goheen^9,10^

*1 Natural Resource Ecology Laboratory, Warner College of Natural Resources, Colorado State University, Fort Collins, CO, USA.*

*2 U.S. Geological Survey, Fort Collins Science Center, Fort Collins, CO, USA.*

*3 Ecosystem Science and Sustainability, Colorado State University, Fort Collins, CO, USA.*

*4 Department of Natural Resources, Isfahan University of Technology, Isfahan, Iran.*

*5 Inland Norway University of Applied Sciences, Department of Forestry and Wildlife Management, Campus Evenstad, Koppang, Norway.*

*6 Research Institute of Wildlife Ecology, University of Veterinary Medicine Vienna, Vienna, Austria.*

*7 Department of Terrestrial Biodiversity, Norwegian Institute for Nature Research, Trondheim, Norway.*

*8 Wildlife Conservation Society, Bronx, New York, USA.*

*9 Department of Natural Resource Ecology & Management, Iowa State University, Ames, IA, USA*

*10 Global Resource Systems, Iowa State University, Ames, IA, USA*

** Corresponding author: saeideh.esmaeili@colostate.edu*


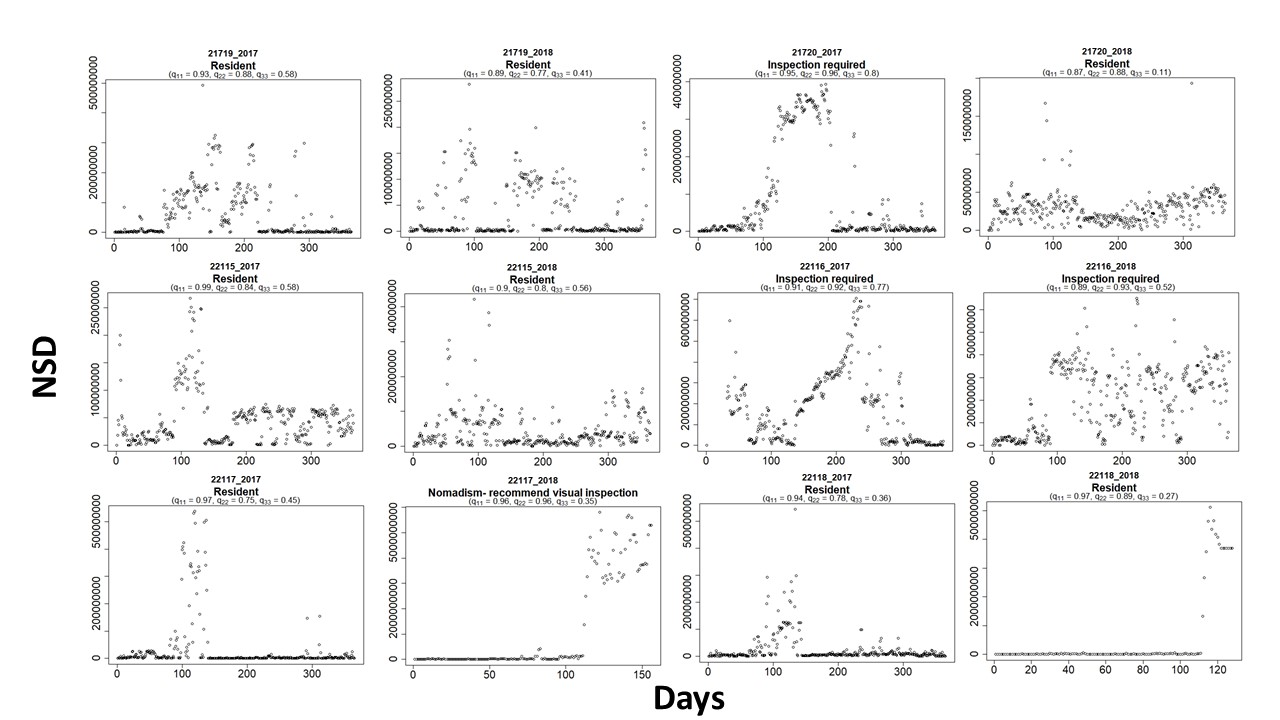


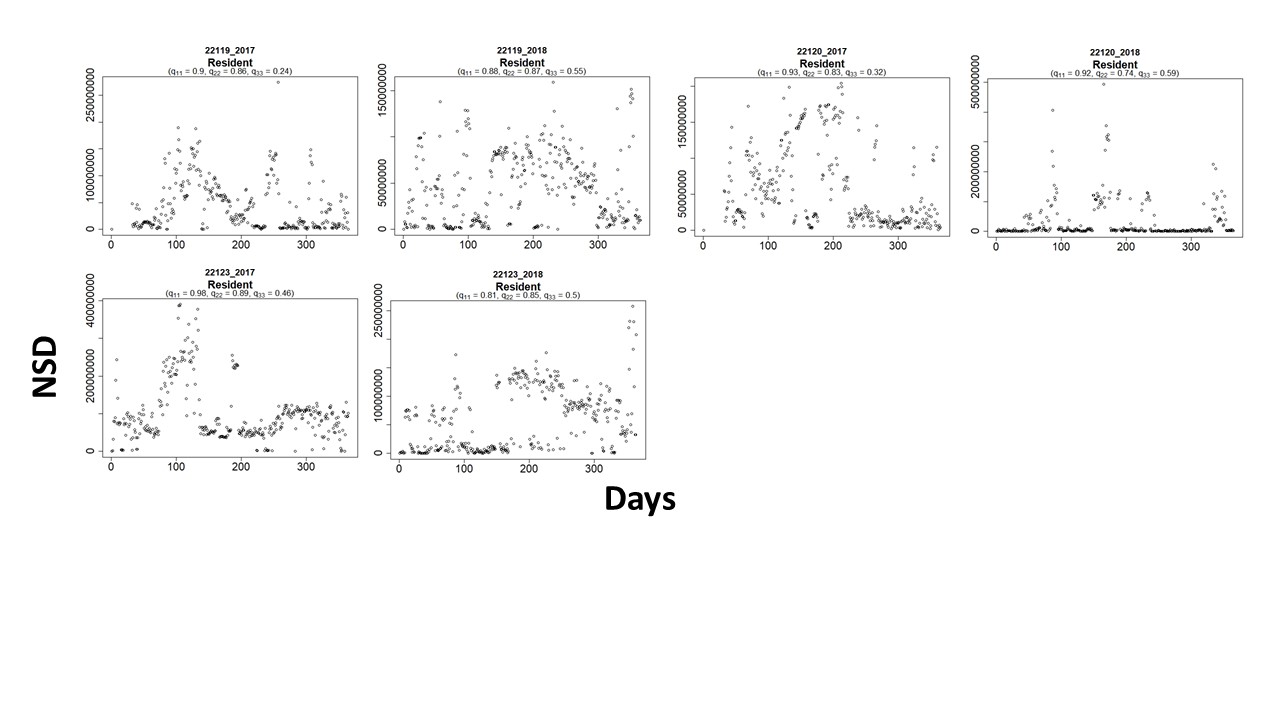


Supplementary Data SD1. Plots of net squared displacement (NSD) values from the annual movement paths of *Equus hemionus onager* plotted against day of year for each animal ID per study year. We used the NSD values and switching probabilities (q-values shown on each plot) to classify movement strategies using the lsmnsd package in R (Bastille-Rousseau et al. 2016). Each plot displays the switching probabilities q₁₁, q₂₂, and q₃₃, along with the assigned movement strategy. We defined a resident strategy as q₂₂ ≤ 0.90 and q₃₃ ≤ 0.90, and a nomadic strategy as q₁₁ > 0.95, q₂₂ > 0.90, and q₃₃ ≤ 0.85. When neither set of criteria was met, we visually interpreted the NSD plots to determine the movement strategy. In the latent state model (Bastille-Rousseau et al. 2016), the transition probabilities q₁₁, q₂₂, and q₃₃ represent the likelihood of an animal remaining in movement states 1, 2, and 3, respectively, from one time step to the next. q₁₁ reflects persistence in a settled or resident state, q₂₂ corresponds to remaining in a seasonal range, and q₃₃ indicates persistence in exploratory or nomadic movement. These probabilities help characterize how consistently an animal maintains each movement behavior over time.

Supplementary Data SD2. Monthly movement-model summaries for nine collared onagers in 2017–2018. For each month (mo) and year (yr), with (n) number of GPS locations, the best-supported continuous-time movement model (movement model) is reported with the effective degrees of freedom for area (area DoF), speed estimate (speed est.; km^2^/day) with lower/upper 95% CIs, home-range area (hr; km²) with lower/upper 95% CIs, home-range crossing time (tau p) with its unit (hours or days), and diffusion rate (km²/day). Home ranges with area DoF < 6 were excluded from inference. See the main manuscript for details of the monthly movement models. OUF = Ornstein–Uhlenbeck Foraging and OU = Ornstein–Uhlenbeck.

| **id** | **yr** | **mo** | **n** | **movement model** | **area DoF** | **speed lower** | **speed_est** | **speed upper** | **hr lower** | **hr est** | **hr upper** | **tau p** | **tau p unit** | **diffusion rate** |
| --- | --- | --- | --- | --- | --- | --- | --- | --- | --- | --- | --- | --- | --- | --- |
| 21719 | 2017 | 1 | 372 | OUF anisotropic | 47.01 | 14.30 | 15.40 | 16.55 | 60.90 | 82.88 | 108.20 | 13.15 | hours | 9.34 |
| 21719 | 2017 | 2 | 336 | OUF anisotropic | 32.32 | 14.22 | 15.27 | 16.37 | 58.52 | 85.38 | 117.24 | 17.52 | hours | 10.02 |
| 21719 | 2017 | 3 | 372 | OUF anisotropic | 13.64 | 18.56 | 19.86 | 21.21 | 221.04 | 408.02 | 651.36 | 1.88 | days | 17.20 |
| 21719 | 2017 | 4 | 360 | OUF anisotropic | 20.10 | 14.31 | 15.15 | 16.02 | 140.65 | 229.95 | 340.84 | 1.19 | days | 12.57 |
| 21719 | 2017 | 5 | 371 | OUF anisotropic | 7.58 | 19.02 | 20.09 | 21.20 | 466.28 | 1110.81 | 2030.26 | 3.28 | days | 27.94 |
| 21719 | 2017 | 6 | 360 | OUF anisotropic | 3.87 | 19.88 | 21.24 | 22.65 | 366.81 | 1386.83 | 3072.59 | 5.64 | days | 21.81 |
| 21719 | 2017 | 7 | 372 | OUF anisotropic | 6.63 | 18.46 | 19.98 | 21.56 | 227.10 | 582.73 | 1103.01 | 3.28 | days | 15.46 |
| 21719 | 2017 | 8 | 372 | OUF anisotropic | 7.81 | 18.67 | 19.79 | 20.94 | 325.77 | 763.91 | 1385.54 | 3.20 | days | 20.18 |
| 21719 | 2017 | 9 | 360 | OUf anisotropic | 91.60 | 13.92 | 14.76 | 15.63 | 28.26 | 35.08 | 42.62 | 2.88 | hours | 12.66 |
| 21719 | 2017 | 10 | 372 | OUF anisotropic | 17.81 | 19.17 | 20.21 | 21.29 | 266.67 | 451.35 | 683.82 | 1.46 | days | 24.66 |
| 21719 | 2017 | 11 | 360 | OUF | 74.99 | 14.75 | 16.05 | 17.36 | 35.43 | 45.05 | 55.80 | 7.27 | hours | 9.66 |
| 21719 | 2017 | 12 | 372 | OUF anisotropic | 41.62 | 12.69 | 13.40 | 14.12 | 64.73 | 89.96 | 119.28 | 13.63 | hours | 10.03 |
| 21719 | 2018 | 1 | 370 | OUF anisotropic | 30.86 | 13.24 | 14.04 | 14.86 | 64.42 | 94.91 | 131.20 | 18.68 | hours | 9.39 |
| 21719 | 2018 | 2 | 366 | OUF anisotropic | 12.58 | 14.71 | 15.58 | 16.48 | 214.83 | 408.40 | 663.10 | 1.92 | days | 14.73 |
| 21719 | 2018 | 3 | 372 | OUF anisotropic | 13.62 | 15.56 | 16.40 | 17.26 | 212.08 | 391.68 | 625.45 | 1.72 | days | 16.85 |
| 21719 | 2018 | 4 | 359 | OUF | 4.94 | 10.71 | 13.77 | 16.82 | 258.52 | 802.74 | 1649.76 | 5.02 | days | 12.11 |
| 21719 | 2018 | 5 | 370 | OUF anisotropic | 24.74 | 15.15 | 16.25 | 17.39 | 80.33 | 124.44 | 178.05 | 23.27 | hours | 10.88 |
| 21719 | 2018 | 6 | 380 | OUF anisotropic | 9.90 | 18.96 | 20.26 | 21.61 | 255.30 | 534.65 | 915.50 | 2.52 | days | 18.34 |
| 21719 | 2018 | 7 | 372 | OUF anisotropic | 16.34 | 18.64 | 19.70 | 20.78 | 222.32 | 386.41 | 595.10 | 1.52 | days | 22.28 |
| 21719 | 2018 | 8 | 372 | OUF anisotropic | 20.33 | 16.27 | 17.22 | 18.20 | 164.92 | 268.78 | 397.59 | 1.34 | days | 15.91 |
| 21719 | 2018 | 9 | 360 | OUF anisotropic | 30.24 | 14.45 | 15.26 | 16.09 | 101.27 | 149.85 | 207.78 | 19.45 | hours | 13.64 |
| 21719 | 2018 | 10 | 372 | OUF anisotropic | 71.73 | 14.54 | 15.47 | 16.42 | 39.88 | 51.00 | 63.46 | 7.18 | hours | 11.58 |
| 21719 | 2018 | 11 | 360 | OUF anisotropic | 24.54 | 14.75 | 15.82 | 16.94 | 87.45 | 135.75 | 194.50 | 1.04 | days | 10.69 |
| 21719 | 2018 | 12 | 372 | OUF anisotropic | 6.30 | 10.94 | 11.65 | 12.38 | 173.64 | 459.29 | 881.42 | 4.49 | days | 7.06 |
| 21720 | 2017 | 1 | 372 | OUF anisotropic | 44.52 | 12.12 | 12.92 | 13.74 | 37.06 | 50.90 | 66.91 | 12.99 | hours | 7.87 |
| 21720 | 2017 | 2 | 336 | OUF anisotropic | 33.48 | 14.74 | 16.75 | 18.89 | 41.73 | 60.44 | 82.57 | 15.60 | hours | 7.38 |
| 21720 | 2017 | 3 | 372 | OUF anisotropic | 33.25 | 14.23 | 15.06 | 15.93 | 81.04 | 117.55 | 160.74 | 18.61 | hours | 12.22 |
| 21720 | 2017 | 4 | 359 | OUF anisotropic | 38.08 | 14.42 | 15.26 | 16.13 | 100.61 | 142.11 | 190.67 | 14.27 | hours | 16.43 |
| 21720 | 2017 | 5 | 372 | OUF anisotropic | 10.20 | 12.39 | 15.21 | 18.33 | 42.89 | 88.67 | 150.80 | 1.51 | days | 4.72 |
| 21720 | 2017 | 6 | 359 | OUF anisotropic | 99.02 | 8.40 | 15.58 | 24.98 | 4.33 | 5.33 | 6.43 | 6.44 | hours | 3.06 |
| 21720 | 2017 | 7 | 372 | OUF anisotropic | 2.17 | 16.63 | 19.18 | 21.91 | 281.33 | 2053.05 | 5547.49 | 16.30 | days | 9.71 |
| 21720 | 2017 | 8 | 372 | OUF | 19.33 | 19.51 | 22.57 | 25.62 | 168.55 | 278.59 | 415.83 | 1.41 | days | 17.12 |
| 21720 | 2017 | 9 | 360 | OUF | 43.72 | 14.74 | 16.12 | 17.49 | 65.79 | 90.64 | 119.42 | 13.28 | hours | 12.33 |
| 21720 | 2017 | 10 | 371 | OUF anisotropic | 24.08 | 13.92 | 14.77 | 15.65 | 100.14 | 156.18 | 224.46 | 1.11 | days | 10.27 |
| 21720 | 2017 | 11 | 360 | OUF anisotropic | 32.79 | 13.03 | 13.88 | 14.75 | 52.41 | 76.24 | 104.46 | 18.15 | hours | 8.55 |
| 21720 | 2017 | 12 | 372 | OUF | 27.35 | 11.82 | 13.11 | 14.40 | 95.80 | 144.93 | 204.08 | 22.90 | hours | 10.53 |
| 21720 | 2018 | 1 | 372 | OUF anisotropic | 28.10 | 13.36 | 14.22 | 15.10 | 70.18 | 105.53 | 147.98 | 21.05 | hours | 9.05 |
| 21720 | 2018 | 2 | 336 | OUF anisotropic | 13.78 | 14.55 | 15.83 | 17.17 | 106.67 | 196.18 | 312.50 | 1.65 | days | 9.13 |
| 21720 | 2018 | 3 | 372 | OUF | 30.57 | 20.92 | 23.27 | 25.61 | 231.41 | 341.60 | 472.91 | 21.04 | hours | 24.06 |
| 21720 | 2018 | 4 | 359 | OUF anisotropic | 40.25 | 21.11 | 23.01 | 24.98 | 120.85 | 168.96 | 225.01 | 14.50 | hours | 18.29 |
| 21720 | 2018 | 5 | 379 | OUF anisotropic | 59.39 | 18.88 | 20.09 | 21.32 | 83.56 | 109.66 | 139.25 | 9.71 | hours | 17.17 |
| 21720 | 2018 | 6 | 359 | OUF anisotropic | 85.08 | 16.80 | 18.01 | 19.27 | 38.74 | 48.49 | 59.33 | 6.00 | hours | 13.48 |
| 21720 | 2018 | 7 | 372 | OUF anisotropic | 104.32 | 16.00 | 17.34 | 18.74 | 26.28 | 32.15 | 38.61 | 5.01 | hours | 10.86 |
| 21720 | 2018 | 8 | 372 | OUF anisotropic | 64.81 | 16.12 | 17.10 | 18.12 | 57.36 | 74.35 | 93.52 | 8.30 | hours | 14.29 |
| 21720 | 2018 | 9 | 360 | OUF anisotropic | 72.12 | 15.07 | 16.00 | 16.96 | 42.37 | 54.14 | 67.33 | 6.80 | hours | 12.36 |
| 21720 | 2018 | 10 | 371 | OUF anisotropic | 82.25 | 17.77 | 19.33 | 20.95 | 43.58 | 54.78 | 67.23 | 7.12 | hours | 12.26 |
| 21720 | 2018 | 11 | 360 | OUF anisotropic | 20.26 | 16.50 | 17.70 | 18.94 | 130.06 | 212.17 | 314.05 | 1.27 | days | 13.17 |
| 21720 | 2018 | 12 | 372 | OUF anisotropic | 95.70 | 18.58 | 21.95 | 25.60 | 28.05 | 34.64 | 41.91 | 6.38 | hours | 11.07 |
| 22115 | 2017 | 1 | 370 | OUF anisotropic | 8.62 | 15.77 | 16.95 | 18.18 | 234.18 | 522.85 | 925.49 | 3.37 | days | 12.22 |
| 22115 | 2017 | 2 | 336 | OUF anisotropic | 19.44 | 14.65 | 15.82 | 17.04 | 76.97 | 127.02 | 189.40 | 1.19 | days | 10.23 |
| 22115 | 2017 | 3 | 372 | OUF anisotropic | 15.98 | 16.14 | 17.05 | 17.98 | 199.22 | 348.65 | 539.20 | 1.45 | days | 16.89 |
| 22115 | 2017 | 4 | 360 | OUF anisotropic | 20.93 | 11.42 | 12.06 | 12.72 | 95.25 | 154.01 | 226.66 | 1.20 | days | 8.92 |
| 22115 | 2017 | 5 | 372 | OUF anisotropic | 19.76 | 17.29 | 18.31 | 19.37 | 173.60 | 285.15 | 423.92 | 1.30 | days | 18.08 |
| 22115 | 2017 | 6 | 359 | OU anisotropic | 5.78 | 0.00 | Inf | Inf | 59.08 | 164.78 | 323.74 | 3.11 | days | 4.76 |
| 22115 | 2017 | 7 | 372 | OUF anisotropic | 58.94 | 23.63 | 28.77 | 34.42 | 70.54 | 92.68 | 117.79 | 10.65 | hours | 16.63 |
| 22115 | 2017 | 8 | 372 | OUF anisotropic | 25.93 | 20.77 | 22.06 | 23.38 | 171.80 | 263.16 | 373.69 | 23.87 | hours | 23.35 |
| 22115 | 2017 | 9 | 360 | OUF anisotropic | 20.00 | 17.89 | 19.33 | 20.82 | 150.86 | 246.99 | 366.44 | 1.31 | days | 13.84 |
| 22115 | 2017 | 10 | 372 | OUF | 18.21 | 16.30 | 18.57 | 20.83 | 170.76 | 287.12 | 433.22 | 1.42 | days | 17.29 |
| 22115 | 2017 | 11 | 360 | OUF | 37.65 | 13.26 | 14.58 | 15.90 | 67.25 | 95.19 | 127.92 | 16.12 | hours | 10.18 |
| 22115 | 2017 | 12 | 372 | OUF | 27.49 | 12.41 | 13.79 | 15.16 | 93.25 | 140.92 | 198.28 | 22.82 | hours | 10.45 |
| 22115 | 2018 | 1 | 369 | OUF anisotropic | 24.88 | 13.12 | 13.93 | 14.77 | 96.02 | 148.54 | 212.34 | 1.05 | days | 9.09 |
| 22115 | 2018 | 2 | 335 | OUF anisotropic | 8.24 | 14.77 | 15.69 | 16.64 | 230.96 | 527.20 | 943.61 | 3.06 | days | 13.63 |
| 22115 | 2018 | 3 | 371 | OUF anisotropic | 14.08 | 17.12 | 18.37 | 19.67 | 183.03 | 334.13 | 529.96 | 1.86 | days | 13.88 |
| 22115 | 2018 | 4 | 359 | OUF anisotropic | 11.12 | 17.46 | 18.45 | 19.47 | 334.11 | 666.31 | 1111.25 | 2.30 | days | 20.64 |
| 22115 | 2018 | 5 | 372 | OUF anisotropic | 18.63 | 16.65 | 17.66 | 18.70 | 131.68 | 219.95 | 330.49 | 1.40 | days | 15.15 |
| 22115 | 2018 | 6 | 360 | OUF anisotropic | 30.56 | 18.01 | 19.18 | 20.39 | 102.79 | 151.75 | 210.08 | 19.66 | hours | 16.34 |
| 22115 | 2018 | 7 | 372 | OUF anisotropic | 62.18 | 19.43 | 21.36 | 23.39 | 66.70 | 86.96 | 109.87 | 10.10 | hours | 14.12 |
| 22115 | 2018 | 8 | 372 | OUF anisotropic | 31.80 | 15.71 | 16.57 | 17.45 | 111.46 | 163.17 | 224.58 | 18.90 | hours | 16.28 |
| 22115 | 2018 | 9 | 359 | OUF anisotropic | 54.65 | 19.42 | 20.67 | 21.95 | 79.30 | 105.37 | 135.08 | 10.04 | hours | 19.33 |
| 22115 | 2018 | 10 | 372 | OUF anisotropic | 44.37 | 18.54 | 19.64 | 20.78 | 105.92 | 145.57 | 191.42 | 13.29 | hours | 18.47 |
| 22115 | 2018 | 11 | 360 | OUF anisotropic | 18.09 | 17.28 | 18.30 | 19.35 | 188.02 | 316.77 | 478.54 | 1.39 | days | 17.78 |
| 22115 | 2018 | 12 | 372 | OUF anisotropic | 6.64 | 13.38 | 14.22 | 15.08 | 194.30 | 498.08 | 942.35 | 3.98 | days | 10.05 |
| 22116 | 2017 | 2 | 334 | OUF anisotropic | 30.03 | 22.56 | 23.96 | 25.40 | 201.84 | 299.09 | 415.16 | 18.07 | hours | 28.34 |
| 22116 | 2017 | 3 | 367 | OUF anisotropic | 3.58 | 14.01 | 15.18 | 16.40 | 190.18 | 771.93 | 1753.55 | 6.76 | days | 8.63 |
| 22116 | 2017 | 4 | 355 | OUF anisotropic | 9.20 | 13.65 | 14.44 | 15.25 | 170.18 | 368.36 | 641.76 | 2.37 | days | 11.80 |
| 22116 | 2017 | 5 | 370 | OUF anisotropic | 8.57 | 17.88 | 19.13 | 20.42 | 249.77 | 559.24 | 991.31 | 2.73 | days | 17.11 |
| 22116 | 2017 | 6 | 360 | OUF anisotropic | 78.24 | 24.24 | 29.01 | 34.20 | 36.89 | 46.65 | 57.54 | 7.77 | hours | 19.71 |
| 22116 | 2017 | 7 | 366 | OUF anisotropic | 91.93 | 18.72 | 21.01 | 23.43 | 30.64 | 38.01 | 46.16 | 6.17 | hours | 12.42 |
| 22116 | 2017 | 8 | 372 | OUF anisotropic | 18.02 | 25.47 | 28.01 | 30.68 | 241.89 | 408.02 | 616.88 | 1.45 | days | 25.41 |
| 22116 | 2017 | 9 | 358 | OUF anisotropic | 17.47 | 28.70 | 31.50 | 34.43 | 386.83 | 658.60 | 1001.49 | 1.50 | days | 33.01 |
| 22116 | 2017 | 10 | 369 | OUF | 10.09 | 18.25 | 21.68 | 25.11 | 463.18 | 962.30 | 1640.81 | 2.73 | days | 26.40 |
| 22116 | 2017 | 11 | 360 | OUF anisotropic | 18.07 | 10.22 | 10.79 | 11.38 | 85.05 | 143.33 | 216.58 | 1.44 | days | 7.15 |
| 22116 | 2017 | 12 | 369 | OUF anisotropic | 25.85 | 9.55 | 10.14 | 10.74 | 40.08 | 61.44 | 87.29 | 1.01 | days | 5.54 |
| 22116 | 2018 | 1 | 369 | OUF anisotropic | 20.33 | 6.64 | 7.05 | 7.46 | 21.77 | 35.48 | 52.49 | 1.19 | days | 2.60 |
| 22116 | 2018 | 2 | 344 | OUF anisotropic | 3.96 | 13.91 | 15.38 | 16.92 | 145.88 | 539.76 | 1186.56 | 5.14 | days | 7.99 |
| 22116 | 2018 | 3 | 370 | OUF anisotropic | 3.66 | 14.78 | 15.57 | 16.39 | 291.41 | 1157.24 | 2608.96 | 4.90 | days | 18.56 |
| 22116 | 2018 | 4 | 358 | OUF anisotropic | 65.08 | 17.42 | 18.70 | 20.04 | 56.68 | 73.42 | 92.31 | 8.14 | hours | 14.47 |
| 22116 | 2018 | 5 | 365 | OUF anisotropic | 3.23 | 22.45 | 23.75 | 25.08 | 1346.65 | 6032.23 | 14163.79 | 8.13 | days | 57.82 |
| 22116 | 2018 | 6 | 356 | OUF anisotropic | 3.88 | 17.72 | 19.03 | 20.37 | 389.27 | 1468.13 | 3249.83 | 6.97 | days | 15.95 |
| 22116 | 2018 | 7 | 370 | OUF anisotropic | 15.48 | 20.90 | 22.23 | 23.61 | 287.11 | 507.70 | 790.13 | 1.69 | days | 23.15 |
| 22116 | 2018 | 8 | 369 | OUF anisotropic | 5.89 | 21.92 | 23.28 | 24.68 | 657.40 | 1812.64 | 3543.41 | 4.86 | days | 28.59 |
| 22116 | 2018 | 9 | 358 | OUF anisotropic | 15.20 | 18.82 | 20.00 | 21.22 | 236.43 | 420.60 | 656.95 | 1.67 | days | 19.72 |
| 22116 | 2018 | 10 | 369 | OUF anisotropic | 4.46 | 20.22 | 21.52 | 22.86 | 646.18 | 2169.87 | 4599.68 | 6.70 | days | 22.22 |
| 22116 | 2018 | 11 | 357 | OUF anisotropic | 39.13 | 25.73 | 27.43 | 29.18 | 227.11 | 319.19 | 426.69 | 14.50 | hours | 38.53 |
| 22116 | 2018 | 12 | 362 | OUF anisotropic | 25.90 | 30.15 | 32.12 | 34.15 | 515.00 | 789.14 | 1120.85 | 23.96 | hours | 66.48 |
| 22117 | 2017 | 1 | 372 | OUF anisotropic | 39.79 | 14.30 | 15.87 | 17.53 | 40.96 | 57.39 | 76.54 | 16.00 | hours | 7.27 |
| 22117 | 2017 | 2 | 336 | OUF | 25.41 | 14.37 | 16.74 | 19.10 | 55.57 | 85.54 | 121.86 | 21.86 | hours | 7.95 |
| 22117 | 2017 | 3 | 372 | OUF anisotropic | 40.46 | 14.54 | 15.37 | 16.22 | 69.22 | 96.69 | 128.68 | 14.43 | hours | 12.52 |
| 22117 | 2017 | 4 | 353 | OUF anisotropic | 15.50 | 22.28 | 23.89 | 25.55 | 247.32 | 437.16 | 680.18 | 1.64 | days | 29.19 |
| 22117 | 2017 | 5 | 372 | OUF anisotropic | 6.92 | 18.02 | 18.99 | 19.98 | 507.91 | 1271.58 | 2379.58 | 3.79 | days | 28.61 |
| 22117 | 2017 | 6 | 360 | OU anisotropic | 204.39 | 0.00 | Inf | Inf | 10.15 | 11.69 | 13.35 | 3.01 | hours | 8.36 |
| 22117 | 2017 | 7 | 370 | OU anisotropic | 131.17 | 0.00 | Inf | Inf | 13.52 | 16.17 | 19.05 | 5.05 | hours | 6.35 |
| 22117 | 2017 | 8 | 371 | OUF anisotropic | 64.46 | 14.60 | 16.01 | 17.48 | 33.91 | 43.98 | 55.35 | 9.68 | hours | 7.91 |
| 22117 | 2017 | 9 | 360 | OUF anisotropic | 93.99 | 16.50 | 18.24 | 20.07 | 27.74 | 34.32 | 41.60 | 5.91 | hours | 9.51 |
| 22117 | 2017 | 10 | 369 | OUF anisotropic | 37.07 | 15.65 | 17.02 | 18.45 | 62.59 | 88.86 | 119.67 | 16.95 | hours | 9.98 |
| 22117 | 2017 | 11 | 360 | OUF anisotropic | 26.04 | 13.22 | 14.04 | 14.88 | 88.60 | 135.59 | 192.41 | 23.89 | hours | 9.32 |
| 22117 | 2017 | 12 | 372 | OUF anisotropic | 40.58 | 13.57 | 14.61 | 15.69 | 42.76 | 59.69 | 79.41 | 14.96 | hours | 8.05 |
| 22117 | 2018 | 1 | 369 | OUF anisotropic | 55.22 | 16.67 | 18.25 | 19.91 | 46.20 | 61.29 | 78.48 | 10.78 | hours | 10.64 |
| 22117 | 2018 | 2 | 336 | OUF anisotropic | 34.54 | 16.30 | 17.85 | 19.47 | 63.62 | 91.58 | 124.55 | 16.60 | hours | 10.67 |
| 22117 | 2018 | 3 | 372 | OUF anisotropic | 26.13 | 23.35 | 24.75 | 26.19 | 262.52 | 401.41 | 569.32 | 23.98 | hours | 30.60 |
| 22117 | 2018 | 4 | 360 | OUF anisotropic | 1.84 | 30.54 | 32.23 | 33.97 | 3079.88 | 29275.95 | 84273.39 | 26.05 | days | 74.36 |
| 22117 | 2018 | 5 | 372 | OUF anisotropic | 25.76 | 33.26 | 35.07 | 36.92 | 648.69 | 995.19 | 1414.65 | 23.23 | hours | 86.61 |
| 22117 | 2018 | 6 |  | OUF anisotropic | 1.62 | 9.36 | 11.03 | 12.83 | 42.01 | 499.21 | 1507.74 | 4.83 | days | 9.09 |
| 22118 | 2017 | 1 | 371 | OUF anisotropic | 31.39 | 13.50 | 14.57 | 15.67 | 55.51 | 81.49 | 112.37 | 20.26 | hours | 8.04 |
| 22118 | 2017 | 2 | 336 | OUF anisotropic | 59.13 | 15.67 | 17.03 | 18.45 | 44.10 | 57.92 | 73.59 | 9.19 | hours | 10.12 |
| 22118 | 2017 | 3 | 370 | OUF anisotropic | 14.80 | 13.95 | 14.81 | 15.69 | 116.34 | 208.81 | 327.86 | 1.53 | days | 11.48 |
| 22118 | 2017 | 4 | 358 | OUF anisotropic | 29.87 | 19.50 | 20.62 | 21.77 | 202.02 | 299.72 | 416.38 | 18.79 | hours | 27.61 |
| 22118 | 2017 | 5 | 372 | OUF anisotropic | 6.04 | 17.00 | 17.95 | 18.94 | 450.59 | 1223.33 | 2375.19 | 4.72 | days | 19.80 |
| 22118 | 2017 | 6 | 360 | OU anisotropic | 169.88 | 0.00 | Inf | Inf | 8.75 | 10.23 | 11.82 | 3.82 | hours | 4.80 |
| 22118 | 2017 | 7 | 372 | OUF anisotropic | 127.12 | 15.37 | 18.14 | 21.14 | 16.18 | 19.41 | 22.93 | 4.45 | hours | 7.08 |
| 22118 | 2017 | 8 | 371 | OUF anisotropic | 53.43 | 17.55 | 18.78 | 20.06 | 77.96 | 103.95 | 133.61 | 11.15 | hours | 13.86 |
| 22118 | 2017 | 9 | 360 | OUF anisotropic | 65.71 | 16.48 | 17.50 | 18.55 | 61.56 | 79.64 | 100.02 | 8.08 | hours | 13.80 |
| 22118 | 2017 | 10 | 372 | OUF anisotropic | 54.20 | 14.43 | 15.39 | 16.38 | 48.24 | 64.17 | 82.35 | 10.87 | hours | 9.83 |
| 22118 | 2017 | 11 | 360 | OUF anisotropic | 52.23 | 12.25 | 13.01 | 13.80 | 40.14 | 53.71 | 69.23 | 10.98 | hours | 7.63 |
| 22118 | 2017 | 12 | 371 | OUF | 38.38 | 12.93 | 14.27 | 15.60 | 54.70 | 77.16 | 103.41 | 16.56 | hours | 8.80 |
| 22118 | 2018 | 1 | 370 | OUF anisotropic | 57.67 | 18.41 | 20.05 | 21.75 | 55.17 | 72.72 | 92.65 | 10.17 | hours | 13.41 |
| 22118 | 2018 | 2 | 336 | OUF anisotropic | 29.08 | 15.03 | 16.33 | 17.68 | 65.92 | 98.37 | 137.21 | 19.58 | hours | 9.70 |
| 22118 | 2018 | 3 | 372 | OUF anisotropic | 36.42 | 21.55 | 23.42 | 25.36 | 100.33 | 142.92 | 192.93 | 16.52 | hours | 19.30 |
| 22118 | 2018 | 4 | 360 | OUF anisotropic | 1.68 | 29.90 | 31.58 | 33.31 | 3231.53 | 35826.18 | 106651.43 | 1.06 | months | 77.75 |
| 22118 | 2018 | 5 | 86 | OUF anisotropic | 2.31 | 5.33 | 6.46 | 7.70 | 6.00 | 39.98 | 105.54 | 2.10 | days | 1.69 |
| 22119 | 2017 | 2 | 332 | OUF anisotropic | 26.73 | 16.90 | 17.99 | 19.11 | 111.94 | 170.27 | 240.63 | 17.92 | hours | 18.41 |
| 22119 | 2017 | 3 | 368 | OUF anisotropic | 13.46 | 16.09 | 17.08 | 18.10 | 167.81 | 311.24 | 498.24 | 1.61 | days | 16.26 |
| 22119 | 2017 | 4 | 358 | OUF anisotropic | 9.11 | 11.06 | 11.71 | 12.38 | 112.57 | 244.83 | 427.59 | 2.53 | days | 7.42 |
| 22119 | 2017 | 5 | 371 | OUF anisotropic | 18.55 | 18.26 | 19.25 | 20.27 | 253.50 | 423.95 | 637.52 | 1.40 | days | 24.71 |
| 22119 | 2017 | 6 | 360 | OUF anisotropic | 65.71 | 20.55 | 22.15 | 23.80 | 54.40 | 70.39 | 88.39 | 8.18 | hours | 21.02 |
| 22119 | 2017 | 7 | 370 | OUF anisotropic | 71.04 | 24.29 | 26.11 | 28.00 | 70.78 | 90.62 | 112.88 | 7.78 | hours | 29.60 |
| 22119 | 2017 | 8 | 372 | OUF anisotropic | 10.48 | 18.26 | 19.34 | 20.45 | 254.37 | 519.82 | 878.52 | 2.05 | days | 20.83 |
| 22119 | 2017 | 9 | 358 | OUF | 7.87 | 14.24 | 17.32 | 20.40 | 262.09 | 612.29 | 1108.53 | 3.06 | days | 17.56 |
| 22119 | 2017 | 10 | 372 | OUF anisotropic | 62.22 | 19.00 | 20.30 | 21.65 | 85.26 | 111.15 | 140.42 | 9.20 | hours | 17.90 |
| 22119 | 2017 | 11 | 360 | OUF anisotropic | 15.40 | 17.12 | 18.53 | 19.99 | 139.06 | 246.30 | 383.67 | 1.67 | days | 13.63 |
| 22119 | 2017 | 12 | 369 | OUF anisotropic | 22.33 | 15.52 | 16.60 | 17.72 | 100.11 | 159.13 | 231.61 | 1.17 | days | 12.95 |
| 22119 | 2018 | 1 | 370 | OUF anisotropic | 26.57 | 15.63 | 16.61 | 17.63 | 102.99 | 156.86 | 221.87 | 23.09 | hours | 16.36 |
| 22119 | 2018 | 2 | 336 | OUF anisotropic | 7.72 | 15.69 | 16.76 | 17.86 | 213.19 | 502.82 | 914.56 | 2.94 | days | 13.10 |
| 22119 | 2018 | 3 | 372 | OUF anisotropic | 14.81 | 16.25 | 17.32 | 18.44 | 178.57 | 320.43 | 503.07 | 1.59 | days | 13.42 |
| 22119 | 2018 | 4 | 359 | OUF | 7.78 | 12.74 | 15.48 | 18.22 | 284.77 | 668.91 | 1214.25 | 2.90 | days | 18.71 |
| 22119 | 2018 | 5 | 369 | OUF anisotropic | 7.44 | 17.00 | 18.34 | 19.74 | 227.17 | 546.54 | 1003.67 | 3.39 | days | 13.02 |
| 22119 | 2018 | 6 | 360 | OUF anisotropic | 10.92 | 17.11 | 18.11 | 19.14 | 288.00 | 578.71 | 969.16 | 2.43 | days | 17.86 |
| 22119 | 2018 | 7 | 370 | OUF anisotropic | 13.77 | 15.97 | 16.84 | 17.73 | 219.68 | 404.16 | 643.95 | 1.92 | days | 17.50 |
| 22119 | 2018 | 8 | 372 | OUF anisotropic | 11.01 | 16.73 | 17.73 | 18.76 | 235.95 | 472.55 | 789.93 | 2.41 | days | 16.07 |
| 22119 | 2018 | 9 | 360 | OUF anisotropic | 16.78 | 14.80 | 15.64 | 16.51 | 146.10 | 251.82 | 385.86 | 1.52 | days | 13.54 |
| 22119 | 2018 | 10 | 372 | OUF | 10.19 | 11.91 | 14.13 | 16.34 | 177.90 | 367.89 | 625.74 | 2.45 | days | 11.75 |
| 22119 | 2018 | 11 | 359 | OUF anisotropic | 14.17 | 14.52 | 15.42 | 16.35 | 147.88 | 269.39 | 426.74 | 1.75 | days | 11.88 |
| 22119 | 2018 | 12 | 369 | OUF | 8.82 | 7.75 | 9.32 | 10.88 | 96.70 | 213.46 | 375.65 | 3.12 | days | 5.25 |
| 22120 | 2017 | 2 | 334 | OUF anisotropic | 14.84 | 19.42 | 20.72 | 22.06 | 211.20 | 378.68 | 594.26 | 1.64 | days | 21.52 |
| 22120 | 2017 | 3 | 372 | OUF anisotropic | 20.65 | 17.55 | 18.65 | 19.78 | 182.24 | 295.73 | 436.25 | 1.27 | days | 17.35 |
| 22120 | 2017 | 4 | 358 | OUF anisotropic | 9.75 | 13.33 | 14.38 | 15.47 | 113.49 | 239.37 | 411.42 | 2.29 | days | 8.07 |
| 22120 | 2017 | 5 | 372 | OUF anisotropic | 3.98 | 18.60 | 19.72 | 20.86 | 466.11 | 1718.45 | 3772.76 | 6.74 | days | 21.14 |
| 22120 | 2017 | 6 | 357 | OU anisotropic | 5.55 | 0.00 | Inf | Inf | 99.28 | 284.50 | 565.48 | 4.49 | days | 5.74 |
| 22120 | 2017 | 7 | 371 | OUF anisotropic | 6.96 | 24.48 | 29.32 | 34.59 | 269.48 | 672.44 | 1256.44 | 3.54 | days | 19.16 |
| 22120 | 2017 | 8 | 372 | OUF anisotropic | 6.52 | 18.46 | 20.00 | 21.60 | 257.10 | 665.86 | 1265.72 | 3.69 | days | 15.00 |
| 22120 | 2017 | 9 | 360 | OUF anisotropic | 23.62 | 20.04 | 22.30 | 24.69 | 153.58 | 240.66 | 346.98 | 1.13 | days | 15.73 |
| 22120 | 2017 | 10 | 365 | OUF anisotropic | 73.23 | 18.21 | 19.57 | 20.97 | 73.85 | 94.17 | 116.93 | 7.82 | hours | 14.10 |
| 22120 | 2017 | 11 | 360 | OUF anisotropic | 28.18 | 16.08 | 17.06 | 18.06 | 128.86 | 193.64 | 271.40 | 20.38 | hours | 14.46 |
| 22120 | 2017 | 12 | 372 | OUF anisotropic | 37.60 | 16.84 | 17.81 | 18.81 | 117.32 | 166.11 | 223.26 | 15.64 | hours | 16.56 |
| 22120 | 2018 | 1 | 369 | OUF anisotropic | 74.92 | 16.58 | 17.73 | 18.92 | 48.28 | 61.39 | 76.05 | 7.59 | hours | 12.45 |
| 22120 | 2018 | 2 | 336 | OUF anisotropic | 29.77 | 18.32 | 19.47 | 20.66 | 151.07 | 224.29 | 311.74 | 18.84 | hours | 19.51 |
| 22120 | 2018 | 3 | 372 | OUF anisotropic | 13.47 | 18.78 | 19.79 | 20.84 | 363.16 | 673.30 | 1077.59 | 1.92 | days | 25.01 |
| 22120 | 2018 | 4 | 360 | OUF anisotropic | 23.84 | 15.48 | 16.33 | 17.20 | 153.62 | 240.16 | 345.73 | 23.11 | hours | 17.75 |
| 22120 | 2018 | 5 | 372 | OUF anisotropic | 32.41 | 18.13 | 19.27 | 20.45 | 139.48 | 203.38 | 279.13 | 17.71 | hours | 16.76 |
| 22120 | 2018 | 6 | 360 | OUF | 11.30 | 17.90 | 21.05 | 24.19 | 402.15 | 796.95 | 1324.48 | 2.29 | days | 26.66 |
| 22120 | 2018 | 7 | 372 | OUF anisotropic | 33.33 | 17.35 | 18.60 | 19.90 | 84.90 | 123.09 | 168.25 | 18.59 | hours | 13.88 |
| 22120 | 2018 | 8 | 372 | OUF anisotropic | 25.12 | 20.68 | 22.16 | 23.69 | 159.72 | 246.52 | 351.86 | 1.04 | days | 19.68 |
| 22120 | 2018 | 9 | 360 | OUF anisotropic | 65.98 | 14.26 | 15.06 | 15.88 | 50.11 | 64.80 | 81.34 | 6.99 | hours | 12.58 |
| 22120 | 2018 | 10 | 371 | OUf | 97.57 | 14.30 | 15.09 | 15.88 | 50.37 | 62.08 | 74.99 | 3.25 | hours | 14.44 |
| 22120 | 2018 | 11 | 359 | OUF anisotropic | 12.71 | 15.02 | 15.85 | 16.71 | 167.49 | 317.13 | 513.75 | 1.55 | days | 15.80 |
| 22120 | 2018 | 12 | 358 | OUF anisotropic | 9.75 | 14.00 | 14.89 | 15.81 | 173.21 | 365.32 | 627.90 | 2.51 | days | 11.13 |
| 22123 | 2017 | 1 | 370 | OUF anisotropic | 13.24 | 16.46 | 17.64 | 18.85 | 171.92 | 320.73 | 515.17 | 2.01 | days | 13.79 |
| 22123 | 2017 | 2 | 335 | OUF anisotropic | 25.56 | 19.49 | 22.91 | 26.62 | 84.01 | 129.12 | 183.78 | 21.70 | hours | 13.04 |
| 22123 | 2017 | 3 | 372 | OUF anisotropic | 31.80 | 16.03 | 16.97 | 17.93 | 108.64 | 159.05 | 218.90 | 18.64 | hours | 16.55 |
| 22123 | 2017 | 4 | 358 | OUF anisotropic | 16.02 | 15.62 | 17.49 | 19.46 | 126.60 | 221.39 | 342.24 | 1.71 | days | 8.91 |
| 22123 | 2017 | 5 | 371 | OUF | 10.30 | 18.81 | 23.93 | 29.04 | 191.96 | 395.17 | 670.49 | 2.66 | days | 12.84 |
| 22123 | 2017 | 6 | 355 | OUF | 6.82 | 9.75 | 20.08 | 30.49 | 64.82 | 163.59 | 307.27 | 3.26 | days | 4.21 |
| 22123 | 2017 | 7 | 371 | OUF anisotropic | 11.72 | 20.84 | 23.32 | 25.94 | 209.10 | 408.35 | 673.16 | 2.20 | days | 15.88 |
| 22123 | 2017 | 8 | 372 | OUF anisotropic | 57.35 | 19.38 | 20.61 | 21.88 | 80.30 | 105.93 | 135.05 | 9.49 | hours | 20.53 |
| 22123 | 2017 | 9 | 359 | OUF anisotropic | 67.10 | 21.29 | 23.99 | 26.85 | 58.25 | 75.15 | 94.16 | 8.56 | hours | 15.95 |
| 22123 | 2017 | 10 | 372 | OUF anisotropic | 78.39 | 20.31 | 22.61 | 25.03 | 60.25 | 76.17 | 93.93 | 7.61 | hours | 14.32 |
| 22123 | 2017 | 11 | 360 | OUF anisotropic | 42.74 | 19.54 | 21.16 | 22.85 | 70.74 | 97.85 | 129.29 | 12.56 | hours | 16.21 |
| 22123 | 2017 | 12 | 372 | OUF anisotropic | 26.87 | 14.99 | 15.83 | 16.69 | 104.84 | 159.26 | 224.87 | 21.42 | hours | 14.74 |
| 22123 | 2018 | 1 | 364 | OUF anisotropic | 22.54 | 18.84 | 20.01 | 21.22 | 139.27 | 220.85 | 320.92 | 1.12 | days | 19.64 |
| 22123 | 2018 | 2 | 336 | OUF anisotropic | 20.95 | 16.80 | 17.91 | 19.05 | 128.68 | 208.01 | 306.10 | 1.13 | days | 15.70 |
| 22123 | 2018 | 3 | 372 | OUF anisotropic | 20.34 | 20.66 | 22.00 | 23.37 | 261.40 | 425.93 | 629.99 | 1.34 | days | 21.78 |
| 22123 | 2018 | 4 | 359 | OUF anisotropic | 35.70 | 14.88 | 15.79 | 16.72 | 98.51 | 140.87 | 190.70 | 16.37 | hours | 13.24 |
| 22123 | 2018 | 5 | 371 | OUF anisotropic | 33.52 | 15.72 | 16.71 | 17.73 | 83.47 | 120.88 | 165.10 | 16.69 | hours | 13.19 |
| 22123 | 2018 | 6 | 360 | OUF anisotropic | 25.63 | 18.28 | 19.36 | 20.47 | 127.57 | 195.95 | 278.78 | 22.94 | hours | 19.36 |
| 22123 | 2018 | 7 | 372 | OUF anisotropic | 35.65 | 19.66 | 20.98 | 22.35 | 103.63 | 148.24 | 200.72 | 17.27 | hours | 18.51 |
| 22123 | 2018 | 8 | 370 | OUF anisotropic | 40.79 | 22.31 | 23.60 | 24.91 | 151.94 | 211.93 | 281.73 | 14.62 | hours | 28.10 |
| 22123 | 2018 | 9 | 358 | OUF anisotropic | 66.83 | 22.37 | 23.62 | 24.90 | 113.89 | 147.00 | 184.28 | 7.44 | hours | 30.60 |
| 22123 | 2018 | 10 | 371 | OUF anisotropic | 49.13 | 18.81 | 20.04 | 21.32 | 81.30 | 109.85 | 142.63 | 11.89 | hours | 18.20 |
| 22123 | 2018 | 11 | 360 | OUF anisotropic | 18.63 | 18.10 | 19.31 | 20.56 | 173.66 | 290.08 | 435.88 | 1.38 | days | 16.85 |
| 22123 | 2018 | 12 | 370 | OUF anisotropic | 19.31 | 13.28 | 14.06 | 14.87 | 107.24 | 177.29 | 264.67 | 1.27 | days | 11.95 |


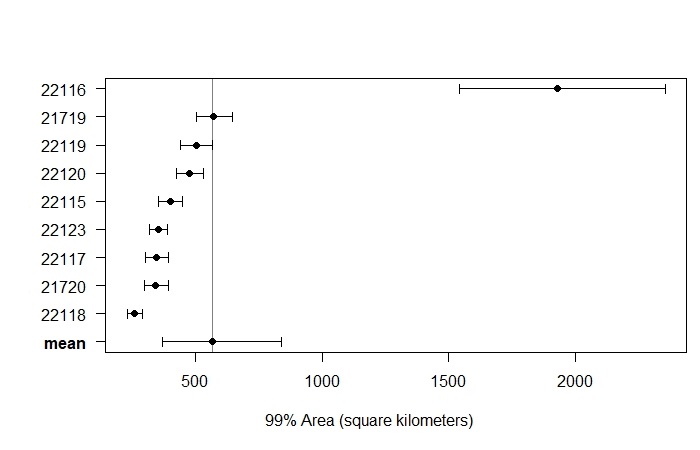


Supplementary Data SD3. Individual home range size estimates (black dots), with 95% confidence intervals (horizontal bars), for nine GPS-collared onagers in south-central Iran during 2017–2018, based on the 99% auto-correlated kernel density estimate. Animal IDs are on the y-axis. The bottom row ("mean") represents the population-level average home range size, calculated using the meta() function in the ctmm package (Fleming and Calabrese 2023).

Supplementary Data SD4. Movement model summaries for nine collared onagers in 2017–2018. For each collared animal (id), the best-supported continuous-time movement model (movement model) is reported with the effective degrees of freedom for area (area DoF), speed estimate (km^2^/day), 95% home-range area (hr; km²) with lower/upper 95% CIs, home-range crossing time (tau p) with its unit, duration of directional persistence (tau v) with its unit, and diffusion rate (km²/day).

| **id** | **movement model** | **area DoF** | **speed_estimate** | **hr lower** | **hr est** | **hr upper** | **tau p** | **tau v** | **diffusion rate** |
| --- | --- | --- | --- | --- | --- | --- | --- | --- | --- |
| 21719 | OUF anisotropic | 239.68 | 19.26 | 389.84 | 444.32 | 502.30 | 2.9 days | 1.06 hours | 15.32 |
| 21720 | OUF anisotropic | 207.72 | 19.73 | 349.95 | 402.88 | 459.48 | 3.36 days | 47.28 minutes | 12.25 |
| 22115 | OUF anisotropic | 280.39 | 20.32 | 324.55 | 366.16 | 410.24 | 2.48 days | 55.13 minutes | 14.80 |
| 22116 | OUF anisotropic | 86.40 | 24.35 | 1314.19 | 1642.09 | 2005.95 | 7.65 days | 56.50 minutes | 22.63 |
| 22117 | OUF | 218.12 | 21.38 | 248.57 | 285.16 | 324.23 | 2.06 days | 46.25 minutes | 13.74 |
| 22118 | OUF anisotropic | 290.60 | 20.35 | 187.91 | 211.53 | 236.53 | 1.52 days | 51.32 minutes | 13.48 |
| 22119 | OUF anisotropic | 236.20 | 20.23 | 396.33 | 452.16 | 511.62 | 2.84 days | 1.02 hours | 16.38 |
| 22120 | OUF anisotropic | 302.50 | 21.37 | 320.91 | 360.38 | 402.11 | 2.22 days | 55.00 minutes | 16.25 |
| 22123 | OUF anisotropic | 382.24 | 22.38 | 277.39 | 307.44 | 339.02 | 1.80 days | 52.48 minutes | 16.84 |

Supplementary Data SD5. Number of GPS locations recorded in cultivated lands and their 50-meter buffer for each collared onager (Animal ID), summarized by month and year, in south-central Iran during 2017–2018.

| Animal ID | Year | Jan | Feb | Mar | Apr | May | Jun | Jul | Aug | Sep | Oct | Nov | Dec |
| --- | --- | --- | --- | --- | --- | --- | --- | --- | --- | --- | --- | --- | --- |
| 21719 | 2017 | 0 | 0 | 0 | 0 | 0 | 0 | 0 | 0 | 0 | 0 | 0 | 0 |
| 21719 | 2018 | 0 | 1 | 0 | 0 | 0 | 0 | 0 | 10 | 0 | 0 | 1 | 0 |
| 21720 | 2017 | 21 | 70 | 0 | 0 | 0 | 0 | 0 | 0 | 0 | 0 | 4 | 12 |
| 21720 | 2018 | 35 | 39 | 55 | 90 | 56 | 0 | 6 | 33 | 45 | 117 | 84 | 111 |
| 22115 | 2017 | 0 | 6 | 0 | 0 | 0 | 0 | 0 | 0 | 0 | 0 | 0 | 9 |
| 22115 | 2018 | 11 | 3 | 0 | 2 | 0 | 0 | 6 | 17 | 29 | 84 | 22 | 0 |
| 22116 | 2017 | 0 | 0 | 0 | 0 | 2 | 0 | 0 | 0 | 0 | 0 | 0 | 0 |
| 22116 | 2018 | 0 | 0 | 1 | 15 | 43 | 5 | 5 | 0 | 7 | 4 | 77 | 71 |
| 22117 | 2017 | 13 | 52 | 0 | 0 | 0 | 0 | 0 | 0 | 0 | 0 | 1 | 20 |
| 22117 | 2018 | 40 | 47 | 53 | 75 | 74 | 0 | - | - | - | - | - | - |
| 22118 | 2017 | 28 | 64 | 3 | 0 | 1 | 0 | 0 | 0 | 0 | 0 | 3 | 19 |
| 22118 | 2018 | 79 | 46 | 76 | 84 | 3 | - | - | - | - | - | - | - |
| 22119 | 2017 | 0 | 0 | 0 | 0 | 3 | 3 | 0 | 0 | 0 | 0 | 3 | 1 |
| 22119 | 2018 | 5 | 0 | 1 | 0 | 0 | 2 | 4 | 2 | 0 | 4 | 0 | 1 |
| 22120 | 2017 | 0 | 0 | 0 | 0 | 0 | 0 | 0 | 0 | 0 | 0 | 0 | 6 |
| 22120 | 2018 | 0 | 0 | 0 | 2 | 0 | 0 | 2 | 2 | 0 | 0 | 2 | 0 |
| 22123 | 2017 | 0 | 1 | 0 | 0 | 0 | 0 | 0 | 0 | 0 | 0 | 0 | 8 |
| 22123 | 2018 | 9 | 0 | 0 | 0 | 0 | 1 | 6 | 14 | 67 | 101 | 30 | 0 |

Supplementary Data SD6. Model selection results for zero-inflated negative binomial regression models predicting the number of onager incursions into cultivated lands every 8 days in south-central Iran in 2017-2018. Models include combinations of rainfall (s: smoothed using splines), vegetation indices (QNP MSAVI and ΔMSAVI), seasonal effects (sine/cosine of the day of the year), and year as a factor. We compared models using Akaike’s Information Criterion (AIC); ΔAIC indicates the difference from the best-supported model, and weights represent relative support.

| **Model** | **AIC** | **ΔAIC** | **df** | **weight** |
| --- | --- | --- | --- | --- |
| **s(rain 9M) + sin/cos(yday)** | **612.404** | **0.000** | **9** | **0.962** |
| s(rain 12M) + sin/cos(yday) | 618.878 | 6.474 | 8 | 0.038 |
| ΔMSAVI + year | 636.852 | 24.447 | 6 | 0.000 |
| ΔMSAVI + sin/cos(yday) | 639.470 | 27.066 | 7 | 0.000 |
| s(rain 6M) + sin/cos(yday) | 647.735 | 35.330 | 8 | 0.000 |
| s(rain 3M) + sin/cos(yday) | 668.582 | 56.177 | 8 | 0.000 |
| sin/cos(yday) | 670.405 | 58.000 | 7 | 0.000 |
| null | 673.293 | 60.888 | 3 | 0.000 |


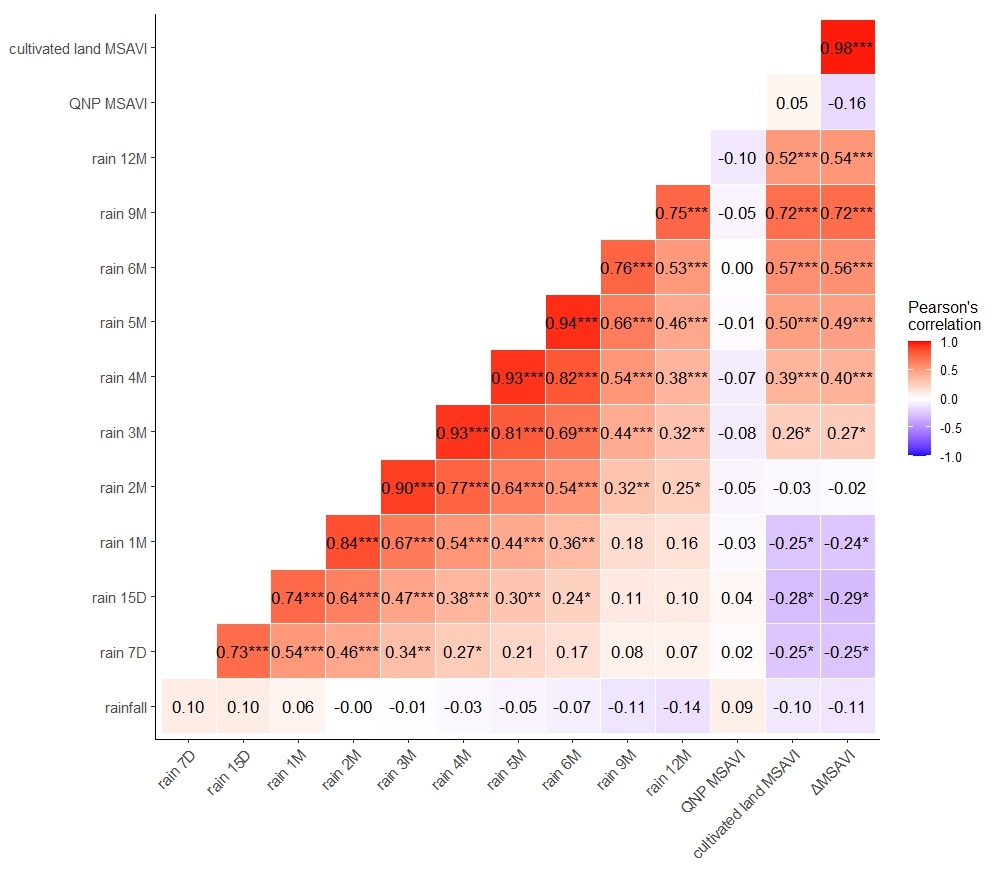
 Supplementary Data SD7. Pearson correlation matrix among vegetation indices and rainfall variables at 8-day intervals. We computed correlations using all pairwise combinations of variables and visualized them using the ggcorrplot package in R (Kassambara & Kassambara 2019). Only the lower triangle of the matrix is shown. Each cell represents the Pearson correlation coefficient, with color intensity indicating the strength and direction of the relationship (red = positive, blue = negative). Asterisks indicate statistical significance (p < 0.05 *, p < 0.01 **, p < 0.001 ***). rainfall: total rainfall during the focal 8-day interval - rain 7D / 15D / 1M / 2M / 3M, 4M, 5M, 6M, 9M, and 12M: cumulative rainfall over the past 7 days, 15 days, 1 month, up to 12 months - cultivated land MSAVI: Modified Soil-Adjusted Vegetation Index (MSAVI) averaged across onager locations within cultivated lands – QNP MSAVI: MSAVI averaged across onager locations within Qatrouiyeh National Park (south-central Iran) - ΔMSAVI: Vegetation contrast index, calculated as cultivated land MSAVI minus QNP MSAVI. To account for multiple testing, p-values were adjusted using the False Discovery Rate (FDR, Benjamini & Hochberg 1995) method implemented in the stats package in R; asterisks are shown only for correlations that remained significant after FDR correction.


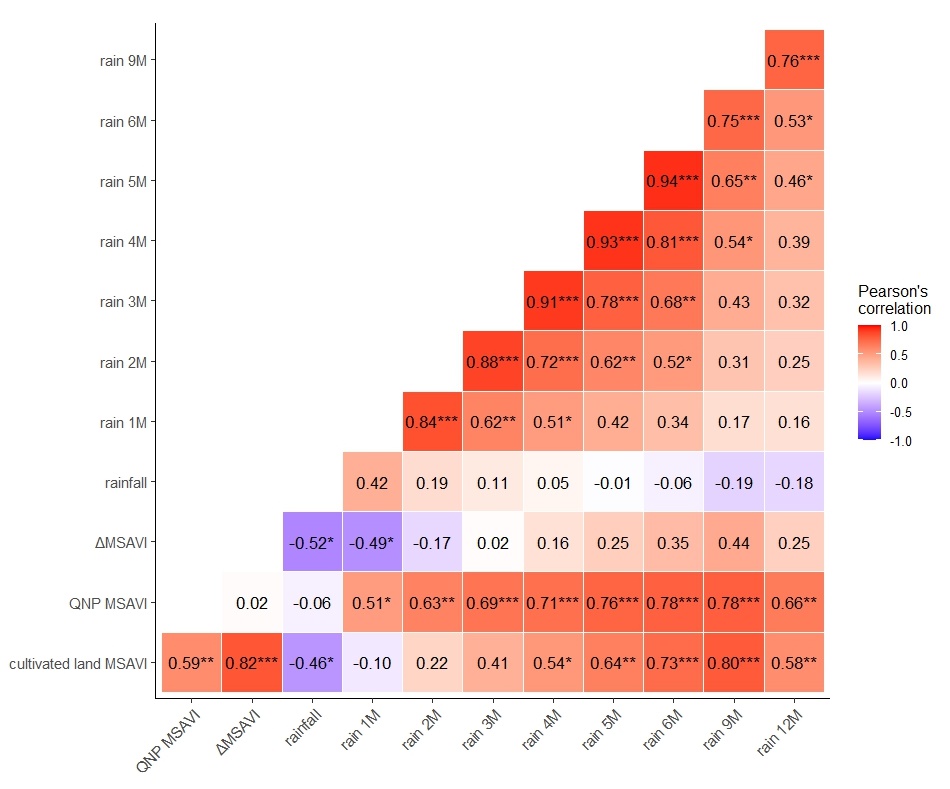


Supplementary Data SD8. Pearson correlation matrix among vegetation indices and rainfall variables at one-month intervals. We computed correlations using all pairwise combinations of variables and visualized them using the ggcorrplot package in R (Kassambara & Kassambara 2019). Only the lower triangle of the matrix is shown. Each cell represents the Pearson correlation coefficient, with color intensity indicating the strength and direction of the relationship (red = positive, blue = negative). Asterisks indicate statistical significance (p < 0.05 *, p < 0.01 **, p < 0.001 ***). rainfall: total rainfall during the focal 1-month interval - rain 1M / 2M / 3M, 4M, 5M, 6M, 9M, and 12M: cumulative rainfall over the past 1 month, up to 12 months - cultivated land MSAVI: Modified Soil-Adjusted Vegetation Index (MSAVI) averaged across onager locations within cultivated lands – QNP MSAVI: MSAVI averaged across onager locations within Qatrouiyeh National Park (south-central Iran) - ΔMSAVI: Vegetation contrast index, calculated as cultivated land MSAVI minus QNP MSAVI. To account for multiple testing, p-values were adjusted using the False Discovery Rate (FDR, Benjamini & Hochberg 1995) method implemented in the stats package in R; asterisks are shown only for correlations that remained significant after FDR correction.

Supplementary Data SD9. Model selection results for generalized linear models predicting the number of onager incursions into cultivated lands every month in south-central Iran in 2017-2018. Models include combinations of rainfall, vegetation indices (QNP MSAVI and ΔMSAVI), seasonal effects (sine/cosine of the month), and year as a factor. We compared models using Akaike’s Information Criterion (AIC); ΔAIC indicates the difference from the best-supported model, and weights represent relative support.

| **Model** | **AIC** | **ΔAIC** | **df** | **weight** |
| --- | --- | --- | --- | --- |
| **rain 9M** | **223.208** | **0.000** | **3** | **0.717** |
| rain 12M | 249.267 | 26.059 | 3 | 0.000 |
| sin(month) + cos(month) | 253.163 | 29.955 | 4 | 0.000 |
| QNP MSAVI | 1409.282 | 1186.074 | 3 | 0.000 |
| rain 6M | 1764.063 | 1540.855 | 3 | 0.000 |
| rain 3M | 2575.979 | 2352.770 | 3 | 0.000 |
| rain 1M | 2768.180 | 2544.972 | 3 | 0.000 |
| rainfall | 2809.339 | 2586.131 | 3 | 0.000 |
| ΔMSAVI | 2866.431 | 2643.223 | 3 | 0.000 |
| null | 2876.305 | 2653.096 | 2 | 0.000 |

**References**

Bastille-Rousseau, G., Potts, J. R., Yackulic, C. B., Frair, J. L., Ellington, E. H., & Blake, S. (2016). Flexible characterization of animal movement pattern using net squared displacement and a latent state model. Movement ecology, 4, 1-12.

Benjamini, Y., and Hochberg, Y. (1995). Controlling the false discovery rate: a practical and powerful approach to multiple testing. Journal of the Royal Statistical Society Series B, 57, 289–300.

Fleming, C. H., & Calabrese, J. M. (2023). ctmm: Continuous-time movement modeling. R package version 1.2, 0. https://cran.r-project.org/web/packages/ctmm/index.html

Kassambara, A., & Kassambara, M. A. (2019). Package ‘ggcorrplot’. R package version 0.1, 3(3), 908.
