# Supplementary material for: When the desert dries: rainfall drives conflicts and conservation challenges for onager (Equus hemionus onager)
Source: J Mammal. 2026 May 1;107(3):462–73. doi: 10.1093/jmammal/gyag017 (PMC13271766; doi:10.1093/jmammal/gyag017)
Supplement: gyag017_Supplementary_Data [file gyag017_supplementary_data.zip › Farsi_abstract_18Feb2026.docx]

**Abstract**

Increasingly, the conservation of large and wide-ranging animals is challenged by environmental variability, static boundaries of protected areas, and the expansion of human activities. The Critically Endangered onager (*Equus hemionus onager*) exemplifies these issues in Qatrouiyeh National Park (QNP) and the surrounding Bahram-e-Goor Protected Area (BPA) in Iran. Using GPS telemetry data from nine adult females tracked over two years, we examined seasonal patterns in movement and incursions into cultivated lands in 2017 and 2018. Net squared displacement analyses indicated that most individuals exhibited range-resident behavior with occasional nomadic movements, with no evidence for migration (i.e., predictable movements to and from distinct seasonal ranges). Both monthly home range size and monthly movement rate varied seasonally, peaking in late spring and early summer (May-July). Individual home ranges were between 257 and 1,928 km², while the extrapolated population-level home range (718 km²; 95% confidence interval: 276–1368) extended well outside QNP, covering large portions of the BPA. Occurrence distributions also expanded beyond the protected area into adjacent cultivated lands, highlighting the use of human-occupied areas by onagers. We recorded 2,285 (out of 72,168) GPS locations within cultivated lands and their surrounding 50-meter buffer, with 60% of these incursions occurring immediately adjacent to QNP. Most incursions occurred at night and were strongly associated with both season and cumulative rainfall over the preceding nine months. These findings emphasize how rainfall-driven variability in resource dynamics shapes the spatial distribution and behavior of onagers, thus elevating the risk of conflict with human. The scale and seasonality of onager movements highlight the need for flexible, landscape-level conservation strategies that extend beyond fixed park boundaries to encompass critical habitats and to mitigate conflict across the broader region.

**Keywords:** Asiatic wild ass, endangered species, Iran, human-occupied landscape, long-distance movement, migration, nomadism, onager, rangeland, wildland-human interface

**وقتی بیابان خشک میشود: بارندگی عامل تعارضات و چالشهای حفاظتی برای گورخر ایرانی (*Equus hemionus onager*) است**

**چکیده**

حفاظت از گونه‌های بزرگ‌جثه و دارای گستره پراکنش وسیع، به‌طور فزاینده‌ای با چالش‌های ناشی از نوسانات محیطی، مرزهای ثابت مناطق حفاظت‌شده و گسترش فعالیت‌های انسانی مواجه است. گورخر ایرانی (Equus hemionus onager) که در فهرست گونه‌های به‌شدت در معرض خطر انقراض (CR) قرار دارد، نمونه‌ای بارز از این چالش‌ها در پارک ملی قطروئیه (QNP) و منطقه‌ حفاظت‌شده‌ بهرام‌گور (BPA) در ایران است. در این پژوهش، با استفاده از داده‌های ردیابی ماهواره‌ای مربوط به ۹ ماده بالغ طی دو سال متوالی، الگوهای فصلی جابه‌جایی و ورود به اراضی کشاورزی در سال‌های ۲۰۱۷ و ۲۰۱۸ بررسی شد. نتایج تحلیل Net Squared Displacement (NSD) رفتار حرکتی افراد نشان داد که اغلب آن‌ها در محدوده‌های ثابت سکونت داشته و تعدادی از آنها جابه‌جایی‌های نامنظم طولانی، بدون شواهدی از مهاجرت منظم میان زیستگاه‌های فصلی، انجام می‌دهند. اندازه‌ محدوده‌ خانگی و نرخ جابه‌جایی ماهانه، هر دو دارای تغییرات فصلی بودند و بیشترین مقدار آن‌ها در اواخر بهار و اوایل تابستان (ماه‌های مه تا ژوئیه) مشاهده شد. محدوده‌ خانگی هر فرد بین ۲۵۷ تا ۱۹۲۸ کیلومتر مربع متغیر بود، در حالی که برآورد محدوده‌ خانگی در سطح جمعیت (۷۱۸ کیلومتر مربع؛ فاصله‌ی اطمینان ۹۵٪: ۲۷۶ تا ۱۳۶۸) فراتر از مرزهای پارک ملی قطروئیه امتداد یافته و بخش‌های وسیعی از منطقه حفاظت‌شده‌ی بهرام‌گور را دربر می‌گرفت. دامنه‌ پراکنش گورخرها فراتر از محدوده‌ حفاظت‌شده گسترش یافت و به اراضی کشاورزی مجاور رسید که بیانگر استفاده‌ این گونه از نواحی انسانی‌شده است. از مجموع ۷۲٬۱۶۸ موقعیت تله‌متری (GPS)، تعداد ۲٬۲۸۵ موقعیت در اراضی کشاورزی و در حاشیه‌ی ۵۰ متری پیرامون آن‌ها ثبت شد، به‌طوری‌که ۶۰٪ از این ورودها به زمین‌های کشاورزی در مجاورت مستقیم با مرز پارک ملی قطروئیه رخ داده بود. بیشتر این ورودها در شب اتفاق افتاده و با فصل و مجموع بارش تجمعی در نه ماه گذشته ارتباط معنی‌داری داشت. یافته‌های این پژوهش نشان داد که تغییرپذیری منابع ناشی از نوسانات بارندگی، در شکل‌دهی به الگوهای مکانی و رفتاری گورخر ایرانی نقش داشته و در نتیجه خطر تعارض با انسان را افزایش داده است. مقیاس و الگوی فصلی جابه‌جایی‌های این گونه، بر ضرورت طراحی راهبردهای حفاظتی منعطف در مقیاس سیمای سرزمین تأکید دارد؛ راهبردهایی که فراتر از مرزهای ثابت پارک‌ها، زیستگاه‌های کلیدی را در بر گیرد و به کاهش تعارض میان انسان و گورخر در سطح منطقه‌ای بینجامد.

**کلمات کلیدی:** ایران، تقابل انسان و حیات وحش، جابجایی های طولانی، گورخر آسیایی، چشم اندازهای انسانی ،کوچ، گونه های در معرض انقراض، مهاجرت، مرتع
