# Supplementary material for: When the desert dries: rainfall drives conflicts and conservation challenges for onager (Equus hemionus onager)
Source: J Mammal. 2026 May 1;107(3):462–73. doi: 10.1093/jmammal/gyag017 (PMC13271766; doi:10.1093/jmammal/gyag017)
Supplement: gyag017_Supplementary_Data [file gyag017_supplementary_data.zip › Attached standard file_ _ Final-Manuscript-Review-Form_SE.pdf]

Journal of Mammalogy

FINAL MANUSCRIPT REVIEW

Manuscript Author:________Saeideh Esmaeili___________________

Manuscript Number:_________ JMAMM-2025-126.R1_________________

Return this form within 2 weeks. Your manuscript in the **Journal of Mammalogy** ***likely will be delayed in publication if you fail to approve each point and return this form.***

The Editorial Staff of the *Journal of Mammalogy* needs your help in maintaining the highest standards of presentation and clarity. Remember, the future value of your contribution rests largely with you.

Your paper is scheduled to be published in the issue of *Journal of Mammalogy* noted above, pending
- your approval of my editorial mark-up,
- certification that you have checked carefully references, scientific names, and data accuracy,
- receipt of your revised manuscript in Scholar One.

Please indicate below that you have attended to each point by initialing each item:

__SE___ I approve editorial changes on the attached mark-up or in editor's comments, or I have returned the manuscript with explanations for alternate changes that retain my intended meaning.

__SE___ I checked queries of the Journal Editor and changed items on my revised manuscript where additional information was requested of me.

___SE__ I checked and corrected each citation for completeness and format and have confirmed that citations in the text are in the Literature Cited, and vice versa.

__SE___ I checked accuracy and spelling of each scientific name.

__SE___ I checked accuracy of all data points in text, tables, and figures.

Your signature ___Saeideh Esmaeili_____ _____ Date ____02/17/2026___________

Thank you for your time and attention to these final details. I look forward to seeing your paper published in the *Journal of Mammalogy*.

Please upload this form as Supporting Information when submitted your revised manuscript.

Alternatively, you may return it to jmammal.editorialoffice@jjeditorial.com
